# The Proteobacterial Methanotroph *Methylosinus trichosporium* OB3b Remodels Membrane Lipids in Response to Phosphate Limitation

**DOI:** 10.1128/mbio.00247-22

**Published:** 2022-05-16

**Authors:** Julie Scanlan, Richard Guillonneau, Mark R. Cunningham, Sahanara Najmin, Michaela A. Mausz, Andrew Murphy, Leanne L. Murray, Limei Zhang, Deepak Kumaresan, Yin Chen

**Affiliations:** a School of Life Sciences, University of Warwick, Coventry, UK; b School of Biological Sciences, Queen’s University Belfast, Belfast, UK; c Research Center for Eco-Environmental Sciences, Chinese Academy of Sciences, Beijing, China; Oregon State University

**Keywords:** *Methylosinus*, lipid remodeling, methanotroph

## Abstract

Methane is a potent greenhouse gas in the atmosphere, and its concentration has continued to increase in recent decades. Aerobic methanotrophs, bacteria that use methane as the sole carbon source, are an important biological sink for methane, and they are widely distributed in the natural environment. However, relatively little is known on how methanotroph activity is regulated by nutrients, particularly phosphorus (P). P is the principal nutrient constraining plant and microbial productivity in many ecosystems, ranging from agricultural land to the open ocean. Using a model methanotrophic bacterium, Methylosinus trichosporium OB3b, we demonstrate here that this bacterium can produce P-free glycolipids to replace membrane phospholipids in response to P limitation. The formation of the glycolipid monoglucuronic acid diacylglycerol requires *plcP-agt* genes since the *plcP-agt* mutant is unable to produce this glycolipid. This *plcP-agt*-mediated lipid remodeling pathway appears to be important for *M. trichosporium* OB3b to cope with P stress, and the mutant grew significantly slower under P limitation. Interestingly, comparative genomics analysis shows that the ability to perform lipid remodeling appears to be a conserved trait in proteobacterial methanotrophs; indeed, *plcP* is found in all proteobacterial methanotroph genomes, and *plcP* transcripts from methanotrophs are readily detectable in metatranscriptomics data sets. Together, our study provides new insights into the adaptation to P limitation in this ecologically important group of bacteria.

## INTRODUCTION

Methane is a powerful greenhouse gas, and its atmospheric concentrations have increased 2.5-fold since preindustrial times (1). Worryingly, methane concentrations in the atmosphere have continued to rise at a rate of ~7.6 ppb each year over the past decade (1, 2). Both human activities and natural environment contribute to methane emissions; however, oxidation of methane by microbes is the only known biological sink for this greenhouse gas. It is estimated that microbial methane uptake by soil comprises ~30 Tg yr^−1^, accounting for 5% of annual methane sink globally (1, 2). Methane-oxidizing microbes are widely distributed in the environment, from agricultural soils to natural wetlands and from coastal sediments to deep-sea methane seeps (reviewed in references 3 and 4). As such, aerobic methanotrophs play an important role in mitigating methane emissions before it is released into the atmosphere.

Phylogenetically, aerobic methanotrophs have been found in *Alphaproteobacteria* (*Methylocystaceae* and *Beijerinckiaceae*), *Gammaproteobacteria* (*Methylococcaceae* and *Methylothermaceae*), and *Verrucomicrobia* (*Methylacidiphilaceae*) (3, 5). Traditionally, these aerobic methanotrophs have been classified as type I (*Methylococcaceae* and *Methylothermaceae*), type II (*Methylocystaceae* and *Beijerinckiaceae*), and type III (*Methylacidiphilaceae*) based on contrasting characteristics in physiology, morphology, and membrane ultrastructures, although this rather simplified classification system for aerobic methanotrophs struggles to keep up with the pace of the ever-expanding discovery of novel methanotrophs in diverse habitats (6).

A variety of factors are known to impact methanotroph activities in the environment, including methane and oxygen concentrations, trace metals (e.g., copper which is required for the activity of the membrane-bound particulate methane monooxygenase [pMMO]), temperature, and salinity, as well as changes in land use (reviewed in reference 4). An important, yet largely overlooked, macronutrient for methanotrophs is phosphorus (P). P is a key nutrient for all forms of life, being a major constituent of nucleic acids and membrane lipids. Indeed, phospholipid fatty acid has long been used as an important biomarker for the identification of methanotrophs and quantification of their activity in response to environmental changes (7). However, conflicting results in the literature exist regarding the role of P in methanotroph activity (8). For example, in Dutch drainage ditches, a strong positive correlation between methane oxidation potential and phosphate concentrations has been observed (8). Similarly, in natural forest and paddy soils, phosphate addition significantly enhanced methane oxidation activities (9, 10). However, other reports found no impact or inhibition of P addition on methane oxidation in microcosm experiments (11, 12). Since P is an essential nutrient for all life forms, it is perhaps not surprising that a variety of strategies are adopted by microbes to cope with P limitation, including reduction of the cellular P quota, recycling of P-containing molecules, and an increase in organic and inorganic P uptake (reviewed in reference 13). As such, a mechanistic understanding of how P affects methanotroph activity is needed in order to better understand the role of P on methane oxidation dynamics in the natural environment.

Here, we report the identification of glycoglycerolipids replacing membrane phospholipids in response to P limitation in the model methanotrophic bacterium Methylosinus trichosporium OB3b (14). Such a membrane lipid remodeling process involves the *plcP* and *agt* genes, which appear to be common in proteobacterial methanotrophs. Interestingly, lipid remodeling is important for the bacterium to grow on methane at low P concentrations. Our study thus provides a mechanistic role for how P availability affects methane oxidation.

## RESULTS

### *M. trichosporium* OB3b remodel membrane lipids in response to P limitation.

**FIG 1:**
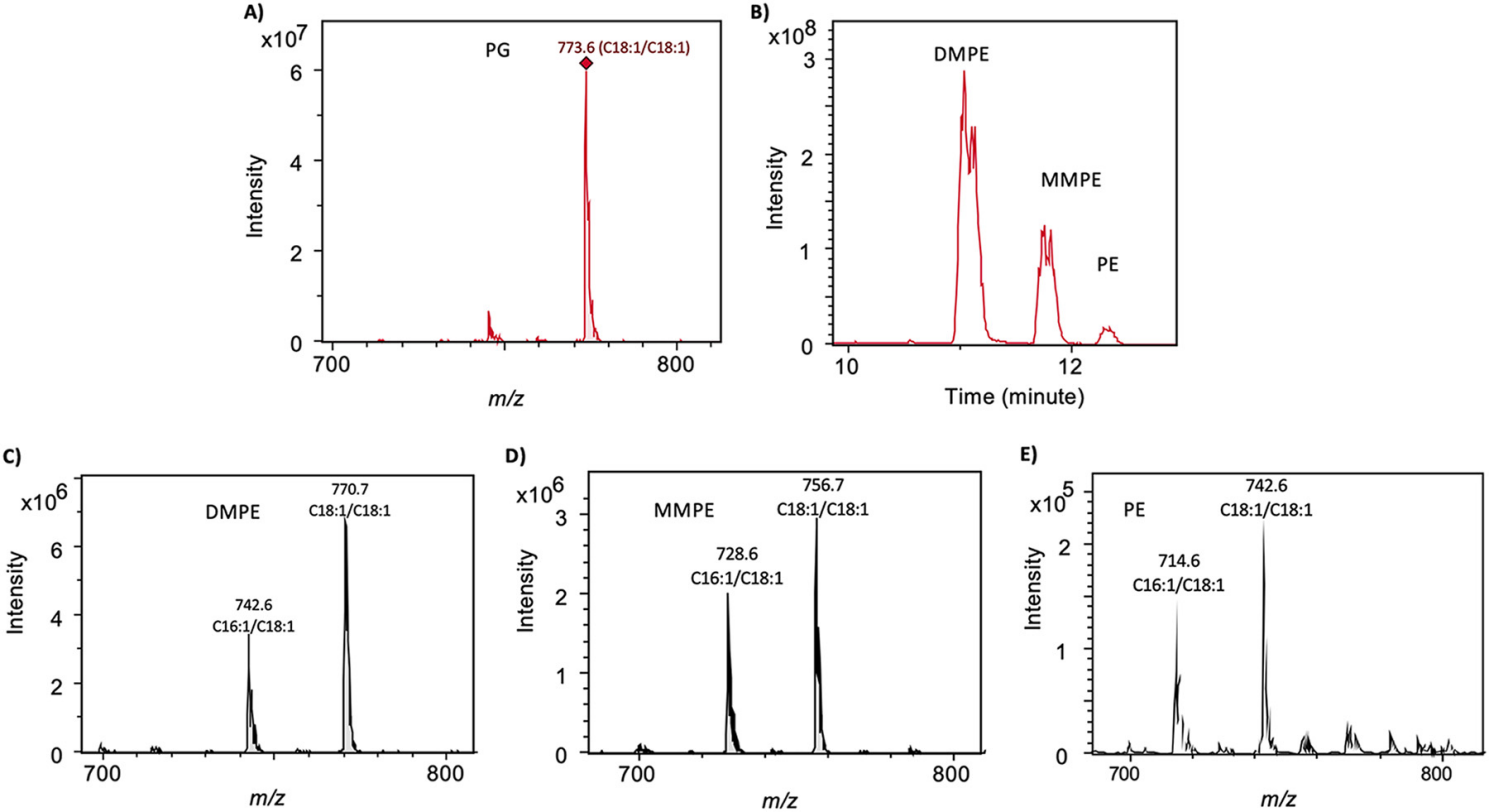
Formation of phospholipids in Methylosinus trichosporium OB3b cultivated in 1× nitrate mineral salts (NMS) medium supplemented with 4 mM phosphate. (A) The phospholipid PG is dominated by one species with an *m/z* of 773.6 that is composed of two monounsaturated fatty acids (C_18:1_/C_18:1_). (B to E) The phospholipid phosphatidylethanolamine (PE) and its methylated derivatives (monomethyl phosphatidylethanolamine, MMPE; dimethyl phosphatidylethanolamine, DMPE) were eluted consecutively between 10 and 13 min, all of which were dominated by two species composed of two monounsaturated fatty acids of C_16:1_/C_18:1_ and C_18:1_/C_18:1_, respectively).

Intact phospholipids in *M. trichosporium* OB3b have previously been analyzed, and it is known that its lipidome is dominated by phospholipids, primarily phosphatidylglycerol (PG) and phosphatidylethanolamine (PE) and its methylated derivatives monomethyl-PE (MMPE) and dimethyl-PE (DMPE) (15, 16). Indeed, when the strain was cultivated under the nitrate mineral salts (NMS) medium, which has 4 mM phosphate, only PG and PE and its methylated derivatives were found. PG is represented by a major species eluted at ~6.1 min with a mass-to-charge ratio (*m/z*) of 773.6 (C_36:2_) in the negative mode ionization (Fig. 1A). When fragmented by MS^n^, this PG species leads to the formation of C_18:1_ fatty acid (see Fig. S1 in the supplemental material), which was common in type II methanotrophs (7). Thus, the bacterium synthesizes a dominant PG lipid consisting of the two monounsaturated fatty acids C_18:1_ and C_18:1_ (C_18:1_/C_18:1_). PE, MMPE, and DMPE were eluted between 11 and 13 min (Fig. 1B). PE and its derivatives had characteristic neutral losses of 141 (PE), 155 (MMPE), and 169 (DMPE), respectively, in the positive ionization mode (see Fig. S2). Each of these lipids are represented by two dominant species that are composed of C_16:1_/C_18:1_ and C_18:1_/C_18:1_ fatty acids, respectively (Fig. 1C to E). Thus, as with PG, PE and its derivatives also appear to be dominated by monounsaturated fatty acid, primarily C_18:1_.

10.1128/mbio.00247-22.1 FIG S1 Fragmentation of the phosphatidylglycerol (PG) lipid *m/z* of 773.6 by MS^n^. Download FIG S1, DOCX file, 0.08 MB. Copyright © 2022 Scanlan et al. 2022 Scanlan et al. https://creativecommons.org/licenses/by/4.0/ This content is distributed under the terms of the Creative Commons Attribution 4.0 International license.

10.1128/mbio.00247-22.2 FIG S2 Fragmentation of the phosphatidylethanolamine (PE), monomethylated PE (MMPE), and demethylated PE (DMPE) by MS^n^. Download FIG S2, DOCX file, 0.4 MB. Copyright © 2022 Scanlan et al. 2022 Scanlan et al. https://creativecommons.org/licenses/by/4.0/ This content is distributed under the terms of the Creative Commons Attribution 4.0 International license.

**FIG 2:**
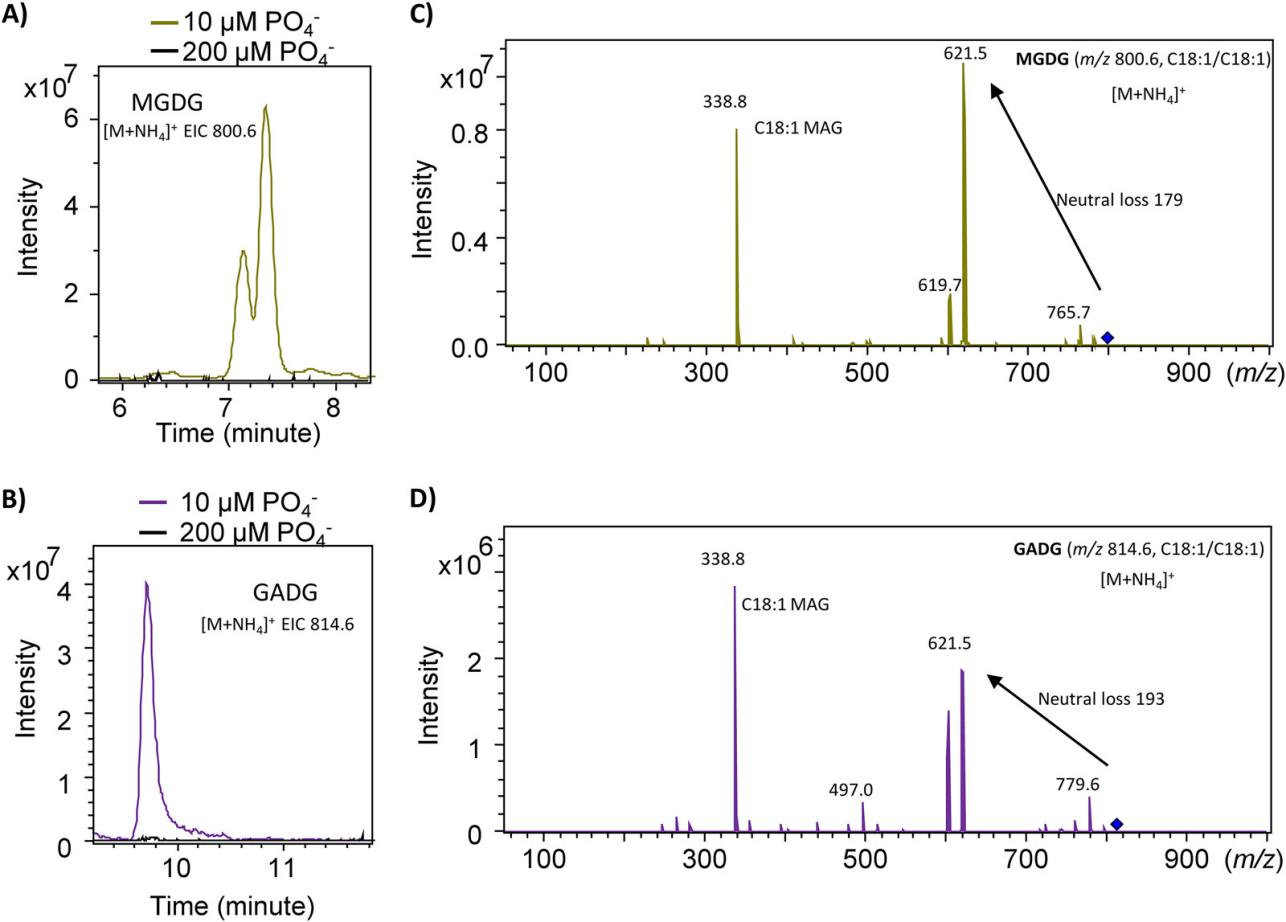
Formation of the new glyceroglycolipids monoglucosyldiacylglycerol (MGDG) and glucuronic acid diacylglycerol (GADG) in response to phosphate limitation in Methylosinus trichosporium OB3b. (A) Formation of the MGDG lipid (extracted ion chromatogram, EIC of the *m/z* 800.6 species, width ± 0.2) showing a characteristic neutral loss of 179 of the hexose (C). (B) Formation of the GADG lipid (extracted ion chromatogram [EIC] of the *m/z* 814.6 species, width ± 0.2) showing a characteristic neutral loss of 193 of the hexuronic acid (D). MS^n^ fragmentation of MGDG and GADG (showing as an ammonium adduct [M+NH_4_]^+^ in the positive mode ionization) produces a monoacylglycerol (MAG) of C_18:1_ (C and D).

To determine the impact of phosphate concentrations on membrane lipids in this methanotroph, we replaced phosphate buffer in the NMS medium with 10 mM HEPES buffer (pH 6.8) and added phosphate back to the medium at a range of concentrations from 10 μM to 4 mM. Diversity of lipids from this bacterium remain largely unchanged when phosphate was supplemented between 50 μM to 4 mM (see Fig. S3), and its lipidome is predominated by PG, PE, and its derivatives (i.e., MMPE and DMPE). However, at 10 μM phosphate in the medium, the intensity of these phospholipids significantly diminished, and new lipids were synthesized (Fig. 2A and B). At approximately 7 to 7.5 min, two major lipids were found with *m/z* values of 800.6 and 772.6, respectively (Fig. 2A). These lipids produce a characteristic neutral loss of 179 in the positive fragmentation by MS^n^ (Fig. 2C), consistent with the monoglucosyldiacylglycerol (MGDG) lipid which was identified previously in other bacteria (see, for example, references 17 and 18). At approximately 9.5 to 10 min, two lipids with *m/z* values of 814.6 and 786.6 were found, which showed a neutral loss of 193 when fragmented in the positive mode (Fig. 2B and D). This is consistent with glucuronic acid diacylglycerol (GADG) lipids that appear to be common surrogate lipids in response to P limitation in other bacteria (17–19). Interestingly, the methanotroph also produced amino-acid-containing ornithine lipids (20), which were eluted at approximately 13 to 13.5 min with characteristic product ions of ornithine lipids (see Fig. S4). Thus, in response to low phosphate in the medium, this methanotrophic bacterium is capable of remodeling its membrane lipids using several surrogate lipids. To the best of our knowledge, these non-P surrogate lipids have not been reported in methanotrophs previously.

10.1128/mbio.00247-22.3 FIG S3 Liquid chromatography-mass spectrometry (LC-MS) chromatogram showing the detection of lipids by MS from Methylosinus trichosporium OB3b cultivated under various phosphate levels (from 4 mM to 10 μM) in the modified NMS medium. Download FIG S3, DOCX file, 0.1 MB. Copyright © 2022 Scanlan et al. 2022 Scanlan et al. https://creativecommons.org/licenses/by/4.0/ This content is distributed under the terms of the Creative Commons Attribution 4.0 International license.

10.1128/mbio.00247-22.4 FIG S4 MS^n^ fragmentation of ornithine lipid produced in Methylosinus trichosporium OB3b in the positive mode ([M+H]^+^, *m/z* 677.6) and negative mode ([M–H]^–^, *m/z* 675.6), respectively. Download FIG S4, DOCX file, 0.1 MB. Copyright © 2022 Scanlan et al. 2022 Scanlan et al. https://creativecommons.org/licenses/by/4.0/ This content is distributed under the terms of the Creative Commons Attribution 4.0 International license.

### The *plcP-agt* genes are involved in glycolipids formation in *M. trichosporium* OB3b.

**FIG 3:**
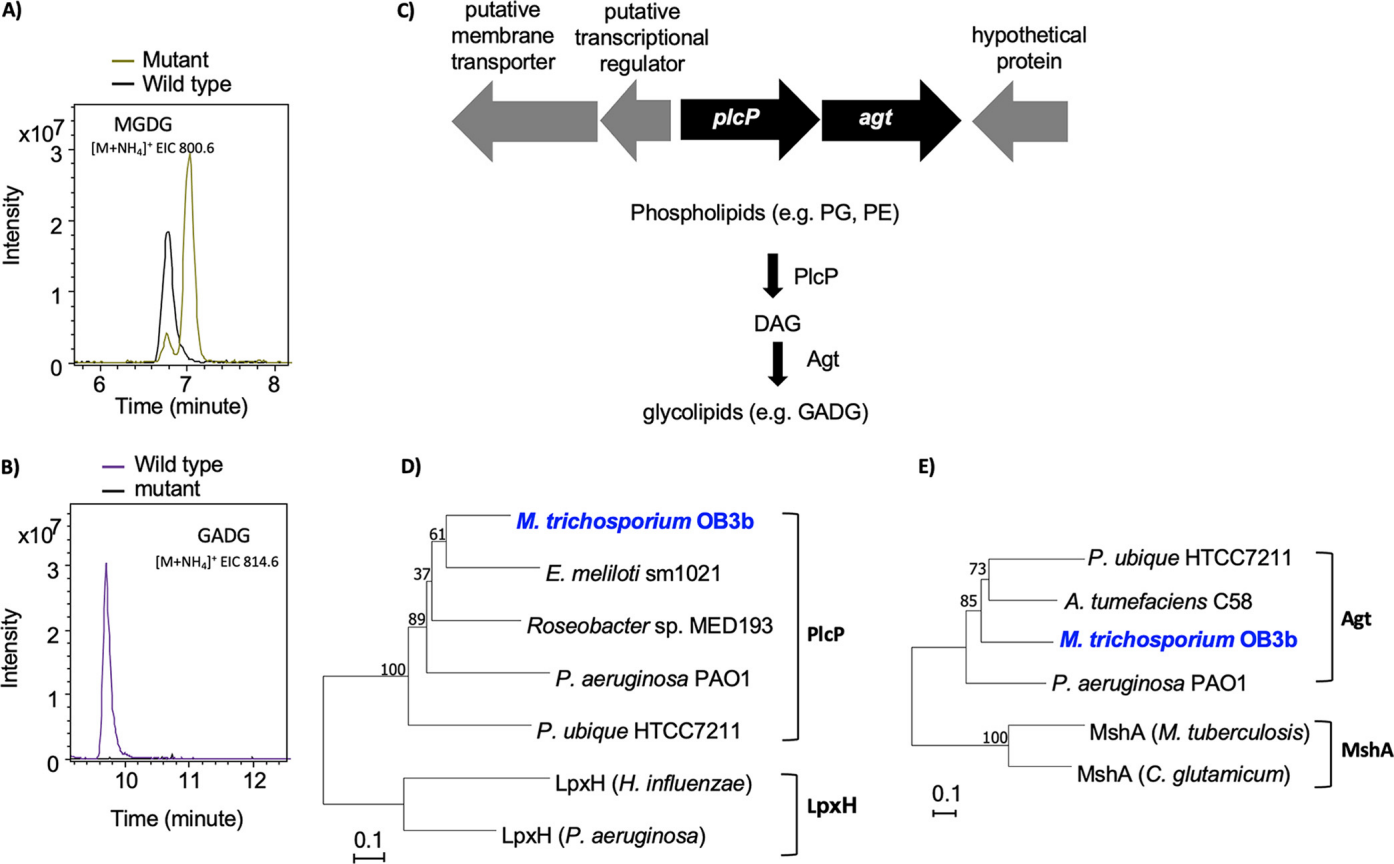
The *plcP-agt* operon in Methylosinus trichosporium OB3b is responsible for the formation of the GADG glycolipids. (A and B) Formation of the glycolipids MGDG (A) and GADG (B) in the wild type, but GADG is not produced in the *plcP-agt* deletion mutant (B). (C) *plcP-agt* genes and their neighborhood and the proposed pathway for lipid remodeling involving PlcP and Agt. DAG, diacylglycerol. (D) Neighbor-joining phylogenetic tree showing PlcP of strain OB3b, together with characterized PlcP from other bacteria, including Ensifer meliloti (24), *Roseobacter* sp. MED193 (18), Pseudomonas aeruginosa (17), and *Pelagibacter ubique* (19). LpxH encoding an enzyme involved in lipid A biosynthesis is used as an outgroup (49, 50). (E) Neighbor-joining phylogenetic tree showing Agt of strain OB3b, together with characterized GT4-group glycosyltransferases, including Agt from *Pelagibacter ubique* (19, 23), Agrobacterium tumefaciens (21), and Pseudomonas aeruginosa (17). MshA involved in mycothiol biosynthesis is used as an outgroup (51, 52).

Formation of MGDG and GADG glycolipids requires a phospholipase C-type protein PlcP and a glycosyltransferase Agt and this PlcP-Agt pathway has been studied in detail in several bacteria, including the marine roseobacters (18, 19), Pseudomonas aeruginosa (17) and Agrobacterium tumefaciens (21). In the genome of strain OB3b, a putative *plcP-agt* cluster was found (Fig. 3). PlcP and Agt of strain OB3b had 51 and 43% sequence identity to the characterized counterparts from *Pelagibacter ubique* HTCC7211, respectively (22, 23). Multiple sequence alignment and phylogenetic analyses of PlcP (Fig. 3D) and Agt (Fig. 3E) from strain OB3b show that they are closely related to the corresponding proteins that are known to be involved in lipid remodeling (17–19, 22, 24). Indeed, key residues that are known to be essential for PlcP and Agt activities are strictly conserved (see Fig. S5), suggesting that PlcP-Agt may encode a functional lipid remodeling pathway in this methanotroph (Fig. 2).

10.1128/mbio.00247-22.5 FIG S5 Sequence alignment of PlcP and Agt. Download FIG S5, DOCX file, 1.5 MB. Copyright © 2022 Scanlan et al. 2022 Scanlan et al. https://creativecommons.org/licenses/by/4.0/ This content is distributed under the terms of the Creative Commons Attribution 4.0 International license.

To confirm the role of *plcP-agt* genes in lipid remodeling in strain OB3b, a knockout mutant was generated using marker exchange mutagenesis. Lipidomics analyses showed that the mutant was unable to produce GADG anymore (Fig. 3), whereas production of MGDG (Fig. 3) and OL (not shown) was not affected. It is therefore likely that an as-yet-unidentified glycosyltransferase may be responsible for the synthesis of MGDG in strain OB3b. Together, the data suggest that the PlcP-Agt pathway in *M. trichosporium* OB3b is responsible for GADG biosynthesis during lipid remodeling in response to P limitation.

### The *plcP-agt* mutant had significant growth defect in phosphorus limitation.

**FIG 4:**
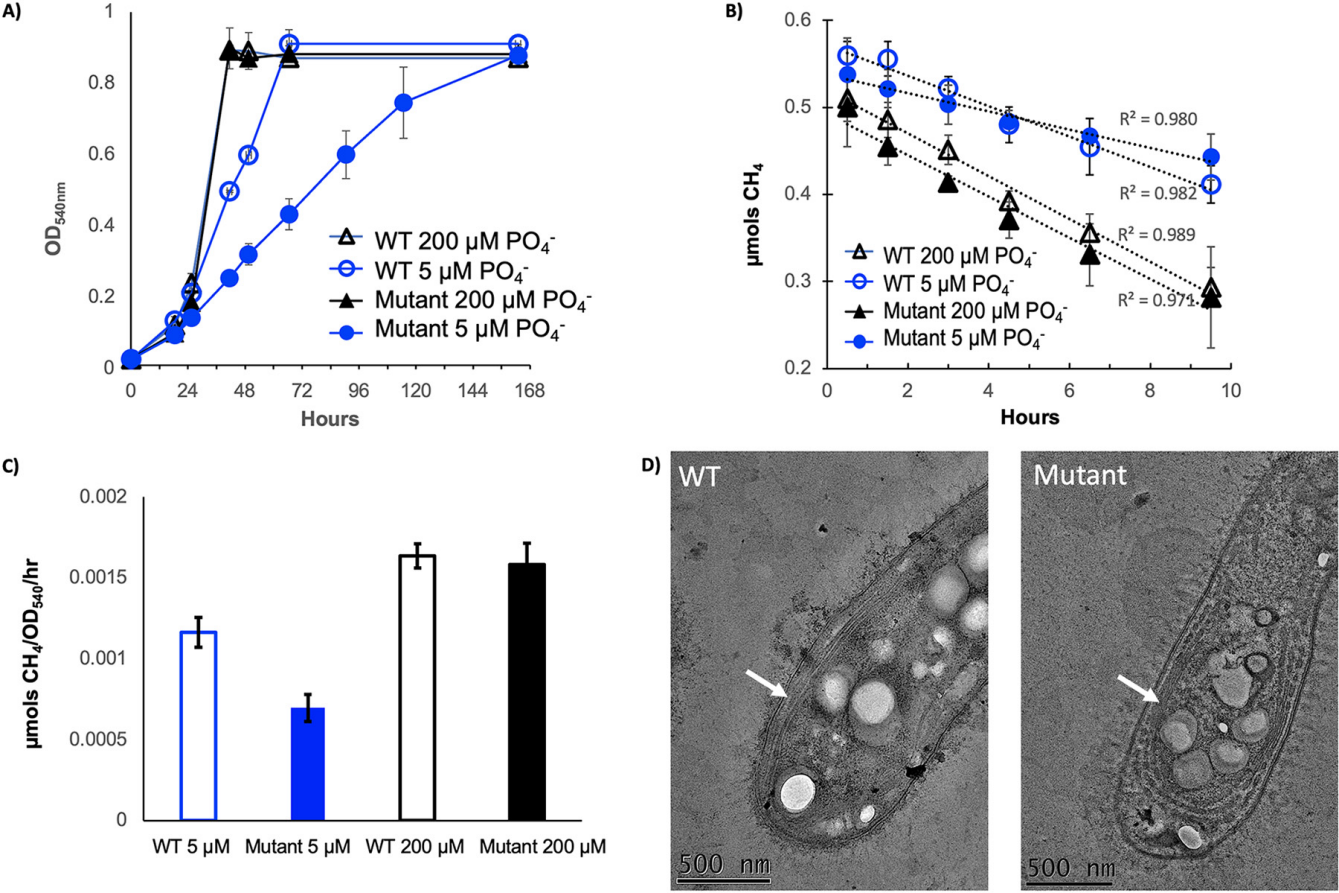
The *plcP-agt* mutant had significant growth defect when cultivated in low-phosphate medium. (A) Growth of the wild type and the mutant under P-replete and P-deplete conditions. All cultures were grown in P-replete medium with 4 mM phosphate and washed once in the medium with no phosphate before inoculating into either P-deplete (5 μM phosphate) or P-replete medium (200 μM phosphate). (B and C) Methane oxidation kinetics (B) and oxidation rates (C) of wild-type and mutant cultivated under P-replete and P-deplete conditions. (D) Thin-section TEM showed significant changes in intracytoplasmic membrane (indicated by white arrows).

To determine the role of lipid remodeling in the growth of *M. trichosporium* OB3b on methane, we cultivated the wild-type strain and the mutant in the modified NMS medium supplied with high (200 μM) and low (5 μM) phosphate and monitored the growth of both strains on methane as the sole carbon source. The data presented in Fig. 4A clearly demonstrate that the ability for lipid remodeling is important for the bacterium to better adapt to phosphate limitation, and no growth defect was observed when the cells were cultivated at higher phosphate concentrations. While no difference in methane oxidation rate was observed for the wild type and the mutant cultivated in the modified NMS medium supplied with high phosphate, the mutant had significantly reduced methane oxidation rate under low-phosphate conditions (Fig. 4B and C). Interestingly, thin-section transmission electron microscopy (TEM) showed that the intracytoplasmic membrane of the mutant cultivated under low-phosphate conditions displayed a noticeable defect in membrane packing, and larger gaps in intracytoplasmic membranes were observed (Fig. 4D), suggesting that the activity of the membrane-bound particulate methane monooxygenase (pMMO) is likely to be affected in the mutant.

### Wide occurrence of lipid remodeling pathway in methanotrophs.

**FIG 5:**
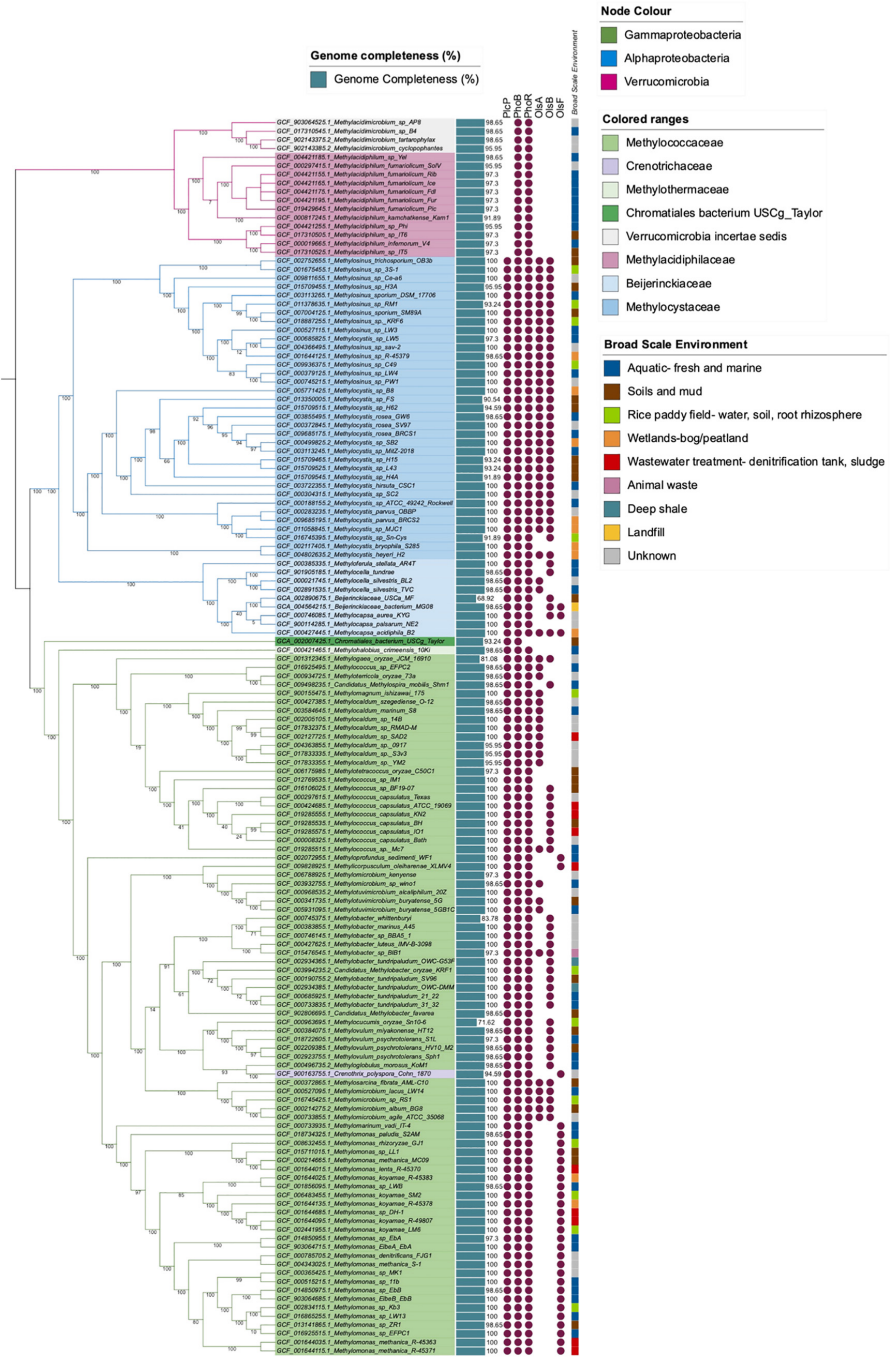
Analysis of lipid remodeling in methanotroph genomes. A phylogenomic tree shows 140 methanotroph genomes and 3 MAGs mapped to the presence or absence of genes involved in *plcP*-mediated lipid remodeling. The tree nodes are colored according to phylum or class (if *Proteobacteria*), the label color ranges indicate bacterial families, and the genome completeness (%) is shown as a blue bar. The broad-scale environment is displayed as a color strip. A purple dot represents a candidate gene has been detected. Bootstrap (1,000 bootstrap alignments applied) values are displayed on the nodes as percentages.

**FIG 6:**
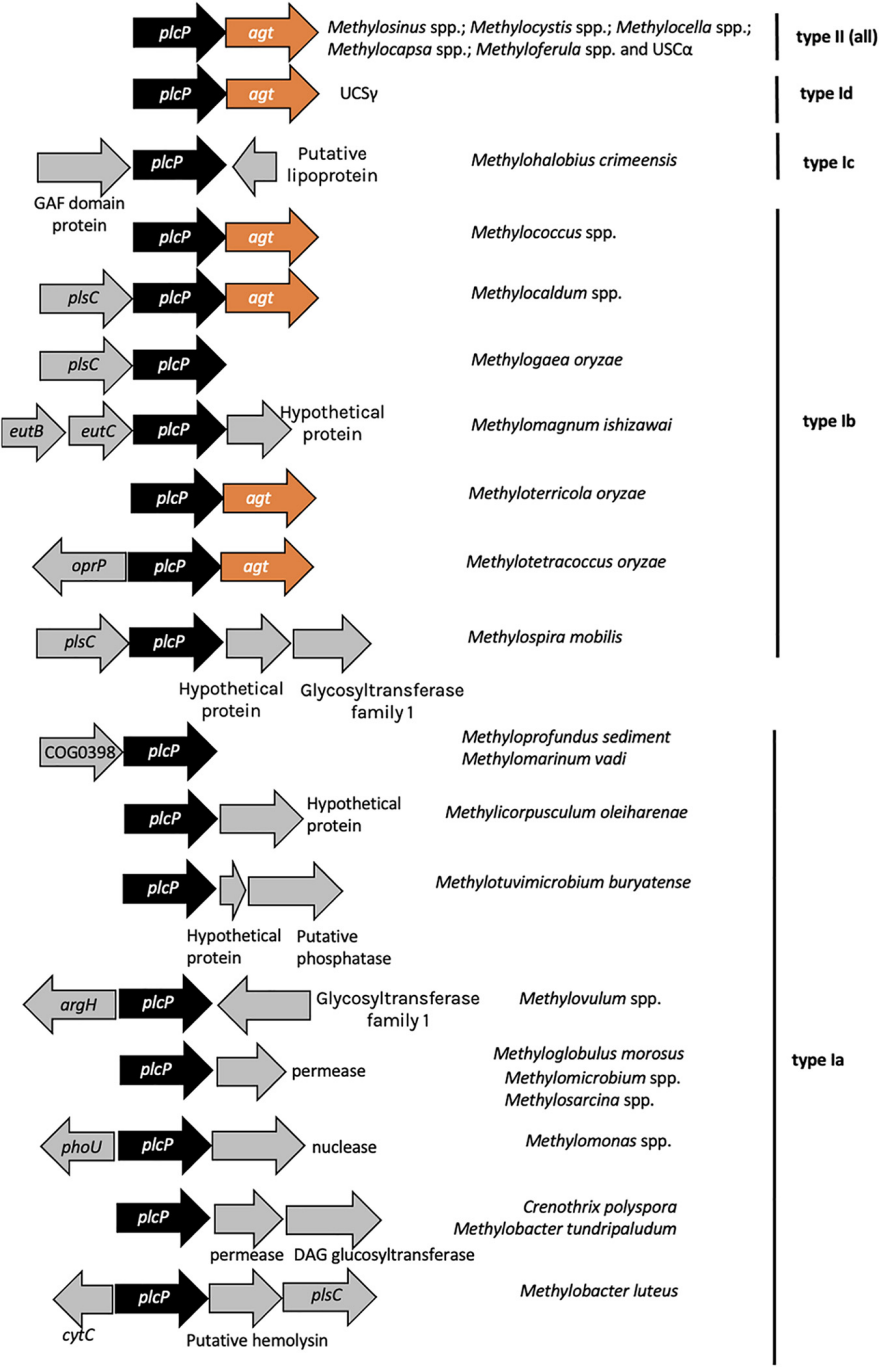
Comparative genomics of PlcP-pathway in methanotrophs showing the neighborhood of *plcP* in type I and type II proteobacterial methanotrophs, including high-affinity methane oxidizers in MAGs.

Having established that PlcP-mediated lipid remodeling is important for this methanotrophic bacterium to better adapt to low-phosphate growth conditions, we used comparative genomics to better understand the distribution of lipid remodeling in genome-sequenced aerobic methanotrophs. We built a profile hidden Markov model using PlcP sequences from characterized bacteria and searched genomes of methanotrophs in the NCBI and JGI-IMG databases (as of November 2021). We mapped out the presence or absence of PlcP in methanotrophs on a phylogenomics tree established using 140 aerobic methanotroph genomes from the RefSeq database and three metagenome assembled genomes of the so-called high-affinity atmospheric methane oxidizers of the Upland Soil Cluster α (USCα) and USCγ. Our data suggest that PlcP appears to be ubiquitous in all proteobacterial methanotrophs, including both type I and type II, whereas the ability to produce ornithine lipids using the OlsB/A pathway is found in some but not all methanotrophs (Fig. 5). Interestingly, PlcP is not found in verrucomicrobial methanotrophs, acidophiles that are isolated from hot and acidic geothermal habitats such as volcano muds (5). In all type II methanotrophs and the type Ib clade, *plcP* is usually located immediately upstream of *agt*, whereas the gene neighborhood of *plcP* in other type I methanotrophs varied significantly (Fig. 6). Another interesting observation is that in the genomes of the USCα and USCγ (25, 26), a similar *plcP-agt* gene cluster was also found, suggesting that these so-called high-affinity methanotrophs may also be capable of lipid remodeling in response to P limitation in the soil.

### Identification of methanotroph *plcP* transcripts in metatranscriptomics data sets.

**FIG 7:**
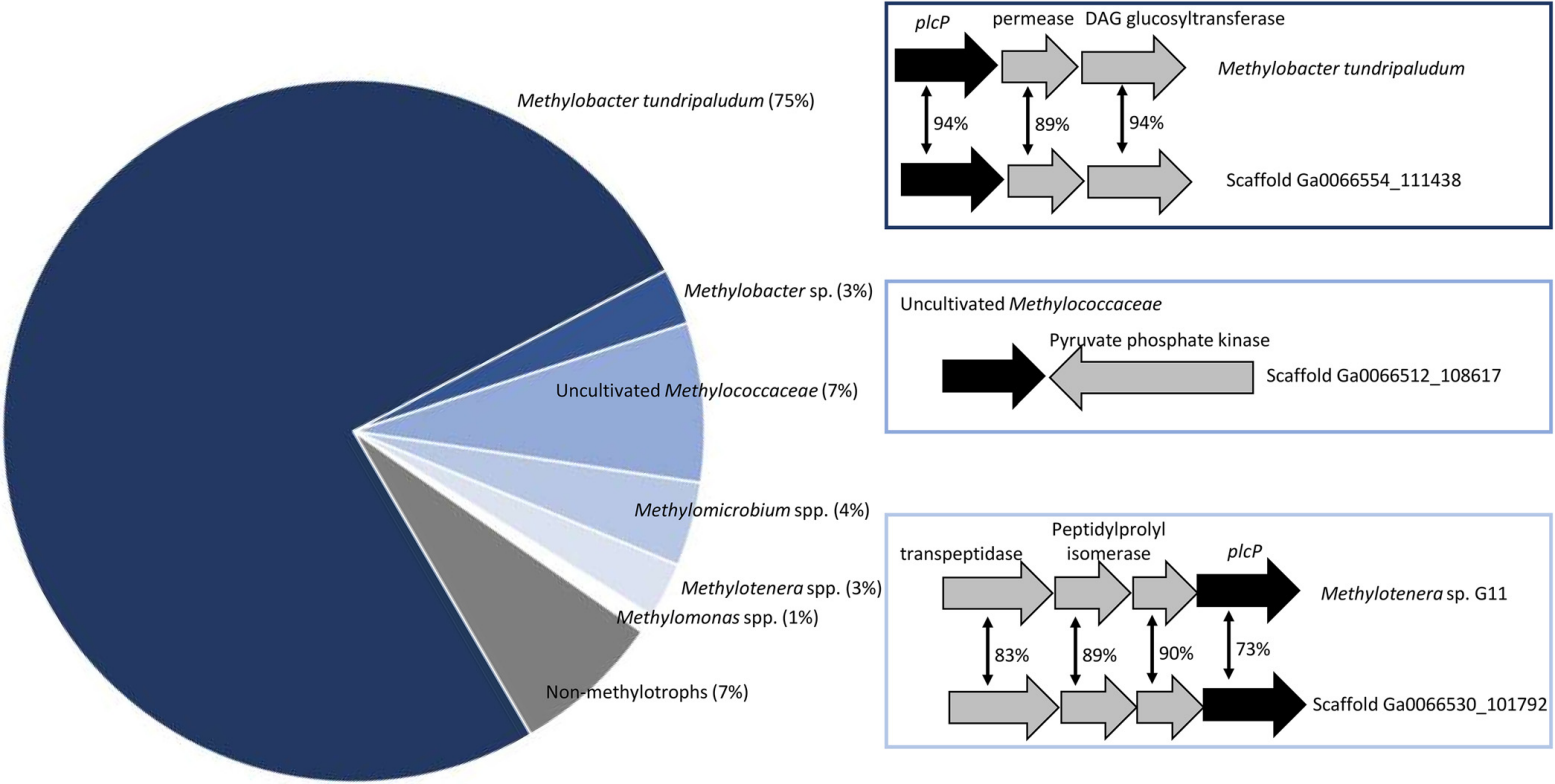
Analysis of metatranscriptomic data sets from Lake Washington sediments. The majority of the PlcP homologs were classified as being from methanotrophs, although PlcP from other bacteria were also found, including the methylotroph *Methylotenera* (3%) and other heterotrophic bacteria (7%). The gene neighborhoods for *plcP* assembled from metatranscriptomics data were analyzed and compared to the closest homologs in genome sequenced bacteria. Bidirectional arrows show protein sequence identity.

To gain further insights into *plcP*-mediated lipid remodeling in environmental samples and to determine whether *plcP* transcripts of methanotrophs can be readily detectable, we analyzed recently published metatranscriptomics data sets from Lake Washington (27). This data set contains comprehensive metatranscriptomics sequencing of microbial communities from the lake sediments and how they responded to oxygen tension in a 14-week period. Furthermore, genome assembly was available, allowing exploitation of gene neighborhood for synteny. We detected 209 PlcP homologs in this data set (Fig. 7), the majority of which were classified as Methylobacter tundripaludum (75%) which is known to be the dominant methanotroph in the lake sediment (27). In scaffolds that were assembled from these metatranscriptomics data sets, *plcP* retrieved from these environmental samples showed conserved gene synteny to that of the genome of Methylobacter tundripaludum and a DAG glucosyltransferase involved in glycolipid production is also found in the environmental scaffold (28). In addition to *Methylobacter* spp., several PlcP homologs were classified as from methanotrophs (e.g., *Methylomicrobium*, *Methylomonas*, or an uncultured *Methylococcaceae* bacterium). Although many of these *plcP* genes were not assembled into contigs, the *plcP* of an uncultured *Methylococcaceae* bacterium was found in Scaffold Ga0066512_108617, and the two genes in this scaffold showed 90 and 92% identity, respectively, to that of an uncultivated *Methylococcaceae* strain from a drinking water metagenome (accession numbers NOU12518.1 and NOU12800.1). Furthermore, *plcP* transcripts from the methylotroph *Methylotenera*, which is also known to be abundant in these lake sediments (29), were detected (Fig. 7).

## DISCUSSION

Here, we demonstrate for the first time that methanotrophs can modulate their membrane lipid compositions in response to the changes of phosphate levels. Formation of new glycolipids appears crucial for the activity of methanotrophs since the mutant lacking the lipid remodeling pathway grew much slower during phosphate limitation. Interestingly, this PlcP-mediated lipid remodeling pathway appears common in proteobacterial methanotrophs of both type I and type II clades but not in the verrucomicrobial clade acidophilic methanotrophs (5).

Inspection of the gene neighborhood provides some interesting insights into the differentiation of proteobacterial methanotrophs. All type II methanotrophs, including *Methylosinus* and *Methylocystis* of the *Methylocystaceae* family and *Methylocella*, *Methylocapsa*, and *Methyloferula* of the *Beijerinckiaceae* family have the glycosyltransferase *agt* gene immediately downstream of the *plcP* gene, suggesting that these type II methanotrophs likely replace membrane phospholipids with glycolipids during phosphate limitation. The same gene organization was also found in the genome sequence of the upland soil clusters USCα and USCγ (25, 26), which can oxidize methane at atmospheric concentrations (~1.8 ppm). However, in type I methanotrophs, the gene neighborhood of *plcP* varied considerably. Although *agt* is commonly located next to *plcP* in *Methylococcus* and *Methylocaldum* spp., no *agt* was found immediately downstream or upstream of type Ia methanotrophs. Instead, in several genomes of type Ia methanotrophs (e.g., *Crenothrix* and Methylobacter tundripaludum), another putative DAG glycosyltransferase is found. Interestingly, *plcP* transcripts from Methylobacter tundripaludum inhabiting lake sediments can be readily detectable, and its gene neighborhood is highly conserved (Fig. 7). In Bacillus subtilis, this DAG glycosyltransferase is involved in the production of a series of glycolipids consisting of mono-, di-, tri-, and tetraglycosyldiacylglycerol (28). However, whether these rather complex lipids can be produced in methanotrophs in the natural environment awaits further experimental validation.

In addition to the glycolipids, strain OB3b also produced an ornithine-containing aminolipid in response to phosphate limitation (see Fig. S3). In proteobacteria, ornithine lipid biosynthesis is carried out by either OlsA/OlsB, encoding a *O*-acyltransferase and a *N*-acyltransferase, respectively, or a bifunctional acyltransferase OlsF (20, 30). The genome of strain OB3b appears to have the OlsA (locus tag Ga0263880_11897)/OlsB (locus tag Ga0263880_112024) pathway for ornithine lipid biosynthesis, although their role in ornithine lipid biosynthesis awaits further experimental validation. Our data suggest that an ornithine lipid is overproduced in P limitation in strain OB3b suggesting that it is involved in substituting membrane phospholipids during P starvation. However, the ecophysiological role of this aminolipid in the membrane of this methanotroph remains to be established.

Many previous studies have focused on the regulation of methanotroph activities in the environment, and it has been firmly established that, among many intrinsically interlinked abiotic factors, substrate availability for methanotrophs, i.e., methane and oxygen, is one of the most influential factors for controlling methanotroph populations in natural ecosystems (see, for example, references 4 and 27). A comprehensive metatranscriptomics analysis of the microbial populations involved in methane cycling in Lake Washington provided new insight into how different methanotroph ecotypes (*Methylobacter* versus *Methylosarcina*) respond to oxygen levels (27, 31). However, studies on how methane oxidation is affected by macronutrients, particularly P, are not always consistent. While many studies have shown that methane oxidation potential can be significantly enhanced by phosphate addition (reviewed in reference 8), others showed no impact of P or indeed an opposite trend, which can be at least partially explained by the fact that methanotrophs were outcompeted by cohabiting microbes (11). To this end, we propose that whether P addition will enhance methanotroph activities in environmental samples will likely be driven by whether or not methanotrophs are truly P limited in the natural environment. Given that *plcP* is conserved in all proteobacterial methanotrophs and that *plcP*-mediated lipid remodeling is induced by P limitation, we propose that *plcP* transcription is likely a reliable biomarker to determine whether P limitation occurs in proteobacterial methanotroph populations in the natural environment. As such, future experiments aiming to link P and methane oxidation activities can benefit from a thorough investigation of *plcP* transcription in methanotrophs.

In summary, we show here that the model proteobacterial methanotroph *M. trichosporium* OB3b can produce glycolipids using the *plcP*-mediated lipid remodeling pathway, which appears to be important for the growth of the bacterium in response to P limitation. The wide occurrence of *plcP* in proteobacterial methanotrophs and the readiness for detection of *plcP* transcripts in metatranscriptomics beg further investigation of its role in methanotroph ecophysiology in the natural environment.

## MATERIALS AND METHODS

### Cultivation of *M. trichosporium* OB3b and its mutant.

*M. trichosporium* was typically cultivated in the NMS medium (32) supplied with 4 mM phosphate (Na_2_HPO_4_-KH_2_PO_4_) as the buffer. To investigate the impact of phosphate concentration on the growth of the bacterium, a modified NMS medium was used where 10 mM HEPES buffer (pH 6.8) was used to replace the phosphate buffer. Phosphate was added to a range of concentrations from 5 μM to 4 mM, and copper was added to a final concentration of 5 μM. Cell growth was monitored by recording the optical density at 540 nm (OD_540_). All growth experiments were set up in triplicate using 250-mL QuickFit flasks containing 50 mL of medium, with an inoculum size of 1 to 2% (vol/vol). The flasks were sealed with Suba seals, with methane added to the headspace in a ratio of 1:5 (methane:air) incubated at 30°C in a shaker (150 rpm).

### Generation of a *plcP-agt* knockout mutant.

The Δ*plcP-agt* mutant was created through homologous recombination using the suicide plasmid pK18mobSacB (33). Two homologous regions flanking the upstream and downstream of the *plcP-agt* gene cluster and a gentamicin (Gm)-resistant gene cassette originated from plasmid p34S-Gm (34) were amplified by PCR and cloned into the pK18mobSacB plasmid using Gibson assembly. The resulting plasmid was mobilized to *M. trichosporium* OB3b through conjugation using Escherichia coli S17.1 λ*pir* as the donor. Transconjugants were selected for on NMS agar plates supplemented with Gm at 2.5 μg/mL, and the mutant was confirmed by PCR amplification and Sanger sequencing. PCR primers used for Gibson cloning and validation of the mutant are presented in Table S1 in the supplemental material.

10.1128/mbio.00247-22.7 TABLE S1 List of primers used in this study. Download Table S1, DOCX file, 0.02 MB. Copyright © 2022 Scanlan et al. 2022 Scanlan et al. https://creativecommons.org/licenses/by/4.0/ This content is distributed under the terms of the Creative Commons Attribution 4.0 International license.

### Complementation of the *plcP-agt* mutant.

The *plcP-agt* mutant was complemented by Gibson cloning of a 2,250-bp fragment containing the *plcP*-*agt* from strain OB3b into the vector pBBR1 carrying kanamycin (Km) resistance (35). This plasmid was mobilized into the existing *plcP-agt* mutant through conjugation as described previously, and transconjugants were selected on NMS plates containing Km (12.5 μg/mL). The complementation was confirmed by PCR amplification using primers plcP-F and Agt R, along with Agt F comp and pK18KmF primers (see Table S1).

### Methane oxidation kinetics.

Wild-type and mutant OB3b (30 mL, OD_540_ ~ 0.5) from P-replete (200 μM phosphate) and P-deplete (5 μM phosphate) medium were harvested from exponentially grown cultures by centrifugation, washed once, and resuspended in 10 mL of the same medium. Resuspended cultures were then put in a 125- mL serum vial, and 0.01% (vol/vol) methane was added. The sample was then left at room temperate (~25°C) to measure methane consumption by gas chromatography (Agilent 6890) with a flame ionization detector fitted with a silica capillary column, as described previously (36). Three biological replicates were used for both wild-type and mutant, and the methane concentrations in the headspace of the serum vials were measured at 0.5, 1.5, 3, 4.5, 6.5, and 9.5 h. Each measurement was carried out twice by injecting 200 μL of gas using a 500-μL Hamilton syringe. Methane oxidation rate was calculated using linear regression analysis in Excel.

### Lipid extraction and lipidomic analyses.

Lipid extraction from *M. trichosporium* OB3b and the Δ*plcP-agt* mutant was carried out in three replicates using a modified Folch extraction method, as described previously (37). Briefly, 1 mL of bacterial culture (OD_540_ = 0.5) was pelleted by centrifugation, and chloroform (1 mL), Milli-Q water (0.3 mL), and methanol (0.5 mL) were then added. The chloroform phase containing the lipids was dried under nitrogen before resuspension in 0.5 to 1 mL of solvent (0.05 mL of 10 mM ammonium acetate in water [pH 9.2] and 0.95 mL acetonitrile). The lipid standard d17:1/12:0 sphingosylphosphoethanolamine (SPE; Avanti Polar Lipids) was added to the samples to a final concentration of 500 nM. Bacterial lipids were separated by a Dionex 3400RS HPLC using an XBridge BEH amide XP column (2.5 μm 3.0 × 150 mm; Waters) on a 15-min gradient from 95% (vol/vol) acetonitrile/5% (wt/vol) ammonium acetate (10 mM [pH 9.2]) to 70% (vol/vol) acetonitrile/30% (wt/vol) ammonium acetate (10 mM [pH 9.2]). The flow rate was 150 μL min^−1^, and the column temperature was 30°C. Ionization and MS fragmentation of lipids were carried out in both positive and negative modes. The drying conditions were 8 L min^−1^ drying gas at 300°C, nebulizing gas pressure of 15 lb/in^2^, and the end cap voltage was 4,500 V in the positive mode and 3,500 V in the negative mode, both with a 500-V offset. Data analyses were carried out using the Bruker Compass Software with DataAnalysis for peak identification and lipid MS^n^ fragmentation and QuantAnalysis for lipid quantification against the internal standard SPE. The retention times of phospholipids PE (~12.2 min) and PG (~6.1 min) and the glycolipid MGDG (~7.1 min) from strain OB3b were confirmed by running authentic lipid standards obtained from Avanti Polar Lipids, Inc. (C_34:0_ PG, 830456P; C_34:0_ PE, 830756P; C_34:1_ MGDG 840522P). The identity of GADG lipid in strain OB3b was further validated by high-resolution MS^n^ using an Orbitrap mass spectrometer (see Fig. S6).

10.1128/mbio.00247-22.6 FIG S6 Complemented mutant restored GADG lipid production under P-deplete but not P-replete conditions (upper panel) and analysis of the GADG lipid [M+NH_4_^+^] *m/z* 814.5 species by high-resolution mass spectrometry using Orbitrap fusion in the positive mode (lower panel). Download FIG S6, DOCX file, 0.3 MB. Copyright © 2022 Scanlan et al. 2022 Scanlan et al. https://creativecommons.org/licenses/by/4.0/ This content is distributed under the terms of the Creative Commons Attribution 4.0 International license.

### Transmission electron microscopy.

Cells of the exponentially growing cultures were collected by centrifugation and fixed with 1% (wt/vol) glutaraldehyde in the NMS medium for 1 h at 4°C. Bacterial pellets were then postfixed with 1% (wt/vol) osmium tetroxide (OsO_4_) in water for 1 h at 4°C. Samples were dehydrated using an increasing ethanol concentration (50 to 100% [vol/vol]) before embedding into low-viscosity resin (Agar Scientific, UK). Ultrathin sections (70 nm) were stained with 2% (wt/vol) uranyl acetate, followed by 3% (wt/vol) lead citrate. The wild-type specimen and the mutant samples cultivated in high and low phosphate were then examined with a transmission electron microscope (Jeol, Tokyo, Japan) operating at 200 kV, equipped with a Gatan OneView IS detector (Gatan Ametek, USA) at the University of Warwick Advanced Bioimaging Research Technology Platform.

### Genomic data acquisition.

A total of 143 known aerobic methanotroph genomes belonging to phyla *Proteobacteria* and *Verrucomicrobia*, available from the National Centre for Biotechnology Information (NCBI) database, were used in this study and included 140 genomes from the RefSeq genome database (38), and three metagenome-assembled genomes (MAGs) representing atmospheric methane oxidizers; *Beijerinckiaceae* bacterium USCα_MF (GCA_002890675.1) (25) and *Chromatiales* bacterium USCg_Taylor (GCA_002007425.1) (26) from the GenBank assembly database (the genome accession numbers are available in Table S2). Metadata from the NCBI was used to identify the source environment and was categorized into broad-scale environments (i.e., aquatic [both fresh and marine], soils and mud, rice paddies, wetlands, wastewater treatment, animal waste, deep shale, and unknown [genomes with no information on the environment]).

10.1128/mbio.00247-22.8 TABLE S2 Genomes of methanotrophic bacteria used in this study. Download Table S2, PDF file, 0.07 MB. Copyright © 2022 Scanlan et al. 2022 Scanlan et al. https://creativecommons.org/licenses/by/4.0/ This content is distributed under the terms of the Creative Commons Attribution 4.0 International license.

### Identification of genes and gene-specific phylogenetic trees.

Genomes were downloaded and concatenated to generate a local methanotroph genomes database using DIAMOND (v0.9.14.115) (39). Validated protein sequences (i.e., *plcP* [WP_024829183.1], lyso-ornithine lipid:acyl-ACP *O*-acyltransferase [*olsA*; AGB69899.1], ornithine:acyl-ACP *N*-acyltransferase [*olsB*; AGB69885.1], bifunctional acyltransferase [*olsF*, A0A6N6NHX3], phosphate response regulator transcription factor [*phoB*; HAI5817470.1], and phosphate regulon sensor protein [*phoR*; P08400]) were used as query sequence to perform Basic Local Alignment Search Tool (BLASTX) searches (1e–10) to detect the presence of genes of interest (39). Homologs retrieved from BLASTX results were translated to amino acid sequences and used in phylogenetic tree construction. Amino acid sequences from methanotroph genomes alongside with other related homologs were used to perform multiple sequence alignment using ClustalO (v 1.2.3) (40). Maximum-likelihood phylogenetic trees were constructed from the multiple sequence alignments using the IQ-Tree2 (v2.1.2) (41) based on the Jones-Taylor-Thornton (JTT) matrix-based model. Bootstrap analysis was performed with 1,000 replicates to provide confidence estimates for phylogenetic tree topologies. Interactive tree of life (ITOL V4) was used for tree visualization annotation with labels colored via taxonomy at the family level (42).

### Phylogenomic analysis.

Phylogenomic relatedness of the methanotroph genomes and MAGs was inferred using 74 single-copy marker genes (specific to the bacterial domain) via the GToTree (v1.5.52) analysis pipeline (43). For each set of protein sequences retrieved using the HMMER3 tool (v3.3.2) using predefined model cutoffs (44), multiple protein sequence alignments were produced using MUSCLE (v3.8.1551) with default settings (45). Automated trimming was performed on the alignments using trimAL (v1.4.rev15) (46). A phylogenetic tree was generated by the concatenated trimmed alignment using FastTree2 (v2.1.10) (47). Taxonomy was assigned using TaxonKit (48). The output newick tree file from GToTree was uploaded to the iTOL platform for further annotation (42). Gene presence-absence data were mapped to the phylogenomic tree to visually represent the detection of *plcP*, *phoB/phoR*, *olsA*, *olsB*, and *olsF* genes in the genomes.

### Metatranscriptomics.

Assembled metatranscriptomes of Lake Washington microbial communities (under the study name Freshwater Sediment Methanotrophic Microbial Communities from Lake Washington under Simulated Oxygen Tension) (27) were analyzed for the presence of PlcP using BLASTP searches carried out at the JGI-IMG site (https://img.jgi.doe.gov). BLASTP searches were carried out with the PlcP of strain OB3b as the query sequence with an e value cutoff of 1e–40. These retrieved 209 PlcP homologs sequences. To further classify the phylogenies of these environmental PlcP homologous, they were then downloaded from IMG, and their identities were classified using a BLASTP search against the NCBI nonredundant protein sequences database (nr). The gene neighborhood of *plcP* from each scaffold was manually inspected using the IMG “gene neighborhoods” function.

## References

[1] IPCC. 2021. AR6 Climate Change 2021: the physical science basis. Contribution of Working Group I to the Sixth Assessment Report of the Intergovernmental Panel on Climate Change. Intergovernmental Panel on Climate Change, Geneva, Switzerland. https://www.ipcc.ch/report/ar6/wg1/.

[2] Jackson RB, Saunois M, Bousquet P, Canadell JG, Poulter B, Stavert AR, Bergamaschi P, Niwa Y, Segers A, Tsuruta A. 2020. Increasing anthropogenic methane emissions arise equally from agricultural and fossil fuel sources. Environ Res Lett 15:e071002. doi:10.1088/1748-9326/ab9ed2.

[3] Knief C. 2015. Diversity and habitat preferences of cultivated and uncultivated aerobic methanotrophic bacteria evaluated based on *pmoA* as molecular marker. Front Microbiol 6:1346. doi:10.3389/fmicb.2015.01346. 26696968 PMC4678205

[4] Bodelier PL, Pérez G, Veraart AJ, Krause SM. 2019. Methanotroph ecology, environmental distribution, and functioning. *In* Methanotrophs, p 1–38. Springer, Cham, Switzerland.

[5] Schmitz RA, Peeters SH, Versantvoort W, Picone N, Pol A, Jetten MSM, Op den Camp HJM. 2021. Verrucomicrobial methanotrophs: ecophysiology of metabolically versatile acidophiles. FEMS Microbiol Rev 45:fuab007. doi:10.1093/femsre/fuab007. 33524112 PMC8498564

[6] Pandit PS, Hoppert M, Rahalkar MC. 2018. Description of ‘*Candidatus* Methylocucumis oryzae’, a novel type I methanotroph with large cells and pale pink colour, isolated from an Indian rice field. Antonie Van Leeuwenhoek 111:2473–2484. doi:10.1007/s10482-018-1136-3. 30066210

[7] Bodelier PLE, Gillisen MJB, Hordijk K, Damste JSS, Rijpstra WIC, Geenevasen JA, Dunfield PF. 2009. A reanalysis of phospholipid fatty acids as ecological biomarkers for methanotrophic bacteria. ISME J 3:606–617. doi:10.1038/ismej.2009.6. 19194481

[8] Veraart AJ, Steenbergh AK, Ho A, Kim SY, Bodelier PLE. 2015. Beyond nitrogen: the importance of phosphorus for CH_4_ oxidation in soils and sediments. Geoderma 259-260:337–346. doi:10.1016/j.geoderma.2015.03.025.

[9] Zhang T, Zhu W, Mo J, Liu L, Dong S. 2011. Increased phosphorus availability mitigates the inhibition of nitrogen deposition on CH_4_ uptake in an old-growth tropical forest, southern China. Biogeosciences 8:2805–2813. doi:10.5194/bg-8-2805-2011.

[10] Lu Y, Wassmann R, Neue H, Huang C. 1999. Impact of phosphorus supply on root exudation, aerenchyma formation and methane emission of rice plants. Biogeochemistry 47:203–218. doi:10.1007/BF00994923.

[11] Alam MS, Xia W, Jia Z. 2014. Methane and ammonia oxidations Interact in paddy soils. Int J Agric Biol 16:365–370.

[12] Zheng Y, Zhang L-M, He J-Z. 2013. Immediate effects of nitrogen, phosphorus, and potassium amendments on the methanotrophic activity and abundance in a Chinese paddy soil under short-term incubation experiment. J Soils Sediments 13:189–196. doi:10.1007/s11368-012-0601-2.

[13] Merchant SS, Helmann JD. 2012. Elemental economy: microbial strategies for optimizing growth in the face of nutrient limitation. Adv Microb Physiol 60:91–210. doi:10.1016/B978-0-12-398264-3.00002-4. 22633059 PMC4100946

[14] Stein LY, Yoon S, Semrau JD, Dispirito AA, Crombie A, Murrell JC, Vuilleumier S, Kalyuzhnaya MG, Op den Camp HJM, Bringel F, Bruce D, Cheng J-F, Copeland A, Goodwin L, Han S, Hauser L, Jetten MSM, Lajus A, Land ML, Lapidus A, Lucas S, Médigue C, Pitluck S, Woyke T, Zeytun A, Klotz MG. 2010. Genome sequence of the obligate methanotroph *Methylosinus trichosporium* strain OB3b. J Bacteriol 192:6497–6498. doi:10.1128/JB.01144-10. 20952571 PMC3008524

[15] Weaver TJ, Patrick MA, Dugan PR. 1975. Whole-cell and membrane lipids of the methylotrophic bacterium *Methylosinus trichosporium*. J Bacteriol 124:602–605. doi:10.1128/jb.124.2.602-605.1975. 810477 PMC235945

[16] Fang J, Barcelona MJ, Semrau JD. 2000. Characterization of methanotrophic bacteria on the basis of intact phospholipid profiles. FEMS Microbiol Lett 189:67–72. doi:10.1111/j.1574-6968.2000.tb09207.x. 10913867

[17] Jones RA, Shropshire H, Zhao C, Murphy A, Lidbury I, Wei T, Scanlan DJ, Chen Y. 2021. Phosphorus stress induces the synthesis of novel glycolipids in *Pseudomonas aeruginosa* that confer protection against a last-resort antibiotic. ISME J 15:3303–3314. doi:10.1038/s41396-021-01008-7. 34031546 PMC8528852

[18] Sebastián M, Smith AF, González JM, Fredricks HF, Van Mooy B, Koblížek M, Brandsma J, Koster G, Mestre M, Mostajir B, Pitta P, Postle AD, Sánchez P, Gasol JM, Scanlan DJ, Chen Y. 2016. Lipid remodelling is a widespread strategy in marine heterotrophic bacteria upon phosphorus deficiency. ISME J 10:968–978. doi:10.1038/ismej.2015.172. 26565724 PMC4796936

[19] Carini P, Van Mooy BA, Thrash JC, White A, Zhao Y, Campbell EO, Fredricks HF, Giovannoni SJ. 2015. SAR11 lipid renovation in response to phosphate starvation. Proc Natl Acad Sci USA 112:7767–7772. doi:10.1073/pnas.1505034112. 26056292 PMC4485111

[20] Vences-Guzmán MÁ, Geiger O, Sohlenkamp C. 2012. Ornithine lipids and their structural modifications: from A to E and beyond. FEMS Microbiol Lett 335:1–10. doi:10.1111/j.1574-6968.2012.02623.x. 22724388

[21] Semeniuk A, Sohlenkamp C, Duda K, Hölzl G. 2014. A bifunctional glycosyltransferase from *Agrobacterium tumefaciens* synthesizes monoglucosyl and glucuronosyl diacylglycerol under phosphate deprivation. J Biol Chem 289:10104–10114. doi:10.1074/jbc.M113.519298. 24558041 PMC3974981

[22] Wei T, Quareshy M, Zhang YZ, Scanlan DJ, Chen Y. 2018. Manganese is essential for PlcP metallophosphoesterase activity involved in lipid remodeling in abundant marine heterotrophic bacteria. Appl Environ Microbiol 84:e01109-18. doi:10.1128/AEM.01109-18. 29802183 PMC6052262

[23] Wei T, Zhao C, Quareshy M, Wu N, Huang S, Zhao Y, Yang P, Mao D, Chen Y. 2021. A glycolipid glycosyltransferase with broad substrate specificity from the marine bacterium “*Candidatus* Pelagibacter sp.” strain HTCC7211. Appl Environ Microbiol 87:e0032621. doi:10.1128/AEM.00326-21. 33931419 PMC8231724

[24] Zavaleta-Pastor M, Sohlenkamp C, Gao JL, Guan Z, Zaheer R, Finan TM, Raetz CR, López-Lara IM, Geiger O. 2010. *Sinorhizobium meliloti* phospholipase C required for lipid remodeling during phosphorus limitation. Proc Natl Acad Sci USA 107:302–307. doi:10.1073/pnas.0912930107. 20018679 PMC2806695

[25] Pratscher J, Vollmers J, Wiegand S, Dumont MG, Kaster AK. 2018. Unravelling the identity, metabolic potential and global biogeography of the atmospheric methane-oxidizing upland soil cluster alpha. Environ Microbiol 20:1016–1029. doi:10.1111/1462-2920.14036. 29314604 PMC6849597

[26] Edwards CR, Onstott TC, Miller JM, Wiggins JB, Wang W, Lee CK, Cary SC, Pointing SB, Lau MCY. 2017. Draft genome sequence of uncultured upland soil cluster *Gammaproteobacteria* gives molecular insights into high-affinity methanotrophy. Genome Announc 5:e00047-17. doi:10.1128/genomeA.00047-17. 28450499 PMC5408097

[27] Zheng Y, Wang H, Yu Z, Haroon F, Hernandez ME, Chistoserdova L. 2020. Metagenomic insight into environmentally challenged methane-fed microbial communities. Microorganisms 8:1614. doi:10.3390/microorganisms8101614. PMC7589939 33092280

[28] Jorasch P, Wolter FP, Zähringer U, Heinz E. 1998. A UDP glucosyltransferase from *Bacillus subtilis* successively transfers up to four glucose residues to 1,2-diacylglycerol: expression of *ypfP* in *Escherichia coli* and structural analysis of its reaction products. Mol Microbiol 29:419–430. doi:10.1046/j.1365-2958.1998.00930.x. 9720862

[29] Chistoserdova L. 2011. Methylotrophy in a lake: from metagenomics to single-organism physiology. Appl Environ Microbiol 77:4705–4711. doi:10.1128/AEM.00314-11. 21622781 PMC3147377

[30] Vences-Guzmán MÁ, Guan Z, Escobedo-Hinojosa WI, Bermúdez-Barrientos JR, Geiger O, Sohlenkamp C. 2015. Discovery of a bifunctional acyltransferase responsible for ornithine lipid synthesis in *Serratia proteamaculans*. Environ Microbiol 17:1487–1496. doi:10.1111/1462-2920.12562. 25040623

[31] Hernandez ME, Beck DA, Lidstrom ME, Chistoserdova L. 2015. Oxygen availability is a major factor in determining the composition of microbial communities involved in methane oxidation. PeerJ 3:e801. doi:10.7717/peerj.801. 25755930 PMC4349146

[32] Whittenbury R, Phillips KC, Wilkinson JF. 1970. Enrichment, isolation and some properties of methane-utilizing bacteria. J Gen Microbiol 61:205–218. doi:10.1099/00221287-61-2-205. 5476891

[33] Schäfer A, Tauch A, Jager W, Kalinowski J, Thierbach G, Puhler A. 1994. Small mobilizable multi-purpose cloning vectors derived from the *Escherichia coli* plasmids pK18 and pK19: selection of defined deletions in the chromosome of *Corynebacterium glutamicum*. Gene 145:69–73. doi:10.1016/0378-1119(94)90324-7. 8045426

[34] Dennis JJ, Zylstra GJ. 1998. Plasposons: modular self-cloning minitransposon derivatives for rapid genetic analysis of gram-negative bacterial genomes. Appl Environ Microbiol 64:2710–2715. doi:10.1128/AEM.64.7.2710-2715.1998. 9647854 PMC106450

[35] Kovach ME, Elzer PH, Hill DS, Robertson GT, Farris MA, Roop IIR, Peterson KM. 1995. Four new derivatives of the broad-host-range cloning vector pBBR1 MCS carrying different antibiotic- resistance cassettes. Gene 166:175–176. doi:10.1016/0378-1119(95)00584-1. 8529885

[36] Chen Y, Dumont MG, McNamara NP, Chamberlain PM, Bodrossy L, Stralis-Pavese N, Murrell JC. 2008. Diversity of the active methanotrophic community in acidic peatlands as assessed by mRNA and SIP-PLFA analyses. Environ Microbiol 10:446–459. doi:10.1111/j.1462-2920.2007.01466.x. 18093158

[37] Silvano E, Yang M, Wolterink M, Giebel HA, Simon M, Scanlan DJ, Zhao Y, Chen Y. 2020. Lipidomic analysis of roseobacters of the pelagic RCA cluster and their response to phosphorus limitation. Front Microbiol 11:552135. doi:10.3389/fmicb.2020.552135. 33408696 PMC7779409

[38] O’Leary NA, Wright MW, Brister JR, Ciufo S, Haddad D, McVeigh R, Rajput B, Robbertse B, Smith-White B, Ako-Adjei D, Astashyn A, Badretdin A, Bao Y, Blinkova O, Brover V, Chetvernin V, Choi J, Cox E, Ermolaeva O, Farrell CM, Goldfarb T, Gupta T, Haft D, Hatcher E, Hlavina W, et al. 2016. Reference sequence (RefSeq) database at NCBI: current status, taxonomic expansion, and functional annotation. Nucleic Acids Res 44:D733–D745. doi:10.1093/nar/gkv1189. 26553804 PMC4702849

[39] Buchfink B, Reuter K, Drost HG. 2021. Sensitive protein alignments at tree-of-life scale using DIAMOND. Nat Methods 18:366–368. doi:10.1038/s41592-021-01101-x. 33828273 PMC8026399

[40] Sievers F, Wilm A, Dineen D, Gibson TJ, Karplus K, Li W, Lopez R, McWilliam H, Remmert M, Söding J, Thompson JD, Higgins DG. 2011. Fast, scalable generation of high-quality protein multiple sequence alignments using Clustal Omega. Mol Syst Biol 7:539. doi:10.1038/msb.2011.75. 21988835 PMC3261699

[41] Nguyen L-T, Schmidt HA, von Haeseler A, Minh BQ. 2015. IQ-TREE: a fast and effective stochastic algorithm for estimating maximum-likelihood phylogenies. Mol Biol Evol 32:268–274. doi:10.1093/molbev/msu300. 25371430 PMC4271533

[42] Letunic I, Bork P. 2019. Interactive Tree of Life (iTOL) v4: recent updates and new developments. Nucleic Acids Res 47:W256–W259. doi:10.1093/nar/gkz239. 30931475 PMC6602468

[43] Lee MD. 2019. GToTree: a user-friendly workflow for phylogenomics. Bioinformatics 35:4162–4164. doi:10.1093/bioinformatics/btz188. 30865266 PMC6792077

[44] Eddy SR. 2011. Accelerated profile HMM searches. PLoS Comput Biol 7:e1002195. doi:10.1371/journal.pcbi.1002195. 22039361 PMC3197634

[45] Edgar RC. 2004. MUSCLE: multiple sequence alignment with high accuracy and high throughput. Nucleic Acids Res 32:1792–1797. doi:10.1093/nar/gkh340. 15034147 PMC390337

[46] Capella-Gutiérrez S, Silla-Martínez JM, Gabaldón T. 2009. trimAl: a tool for automated alignment trimming in large-scale phylogenetic analyses. Bioinformatics 25:1972–1973. doi:10.1093/bioinformatics/btp348. 19505945 PMC2712344

[47] Price MN, Dehal PS, Arkin AP. 2010. FastTree 2: approximate maximum-likelihood trees for large alignments. PLoS One 5:e9490. doi:10.1371/journal.pone.0009490. 20224823 PMC2835736

[48] Shen W, Ren H. 2021. TaxonKit: a practical and efficient NCBI taxonomy toolkit. J Genet Genomics 48:844–850. doi:10.1016/j.jgg.2021.03.006. 34001434

[49] Cho J, Lee M, Cochrane CS, Webster CG, Fenton BA, Zhao J, Hong J, Zhou P. 2020. Structural basis of the UDP-diacylglucosamine pyrophosphohydrolase LpxH inhibition by sulfonyl piperazine antibiotics. Proc Natl Acad Sci USA 117:4109–4116. doi:10.1073/pnas.1912876117. 32041866 PMC7049123

[50] Okada C, Wakabayashi H, Kobayashi M, Shinoda A, Tanaka I, Yao M. 2016. Crystal structures of the UDP-diacylglucosamine pyrophosphohydrase LpxH from *Pseudomonas aeruginosa*. Sci Rep 6:32822. doi:10.1038/srep32822. 27609419 PMC5016852

[51] Newton GL, Koledin T, Gorovitz B, Rawat M, Fahey RC, Av-Gay Y. 2003. The glycosyltransferase gene encoding the enzyme catalyzing the first step of mycothiol biosynthesis (*mshA*). J Bacteriol 185:3476–3479. doi:10.1128/JB.185.11.3476-3479.2003. 12754249 PMC155378

[52] Vetting MW, Frantom PA, Blanchard JS. 2008. Structural and enzymatic analysis of MshA from *Corynebacterium glutamicum*: substrate-assisted catalysis. J Biol Chem 283:15834–15844. doi:10.1074/jbc.M801017200. 18390549 PMC2414306

