# Supplementary material for: The Proteobacterial Methanotroph Methylosinus trichosporium OB3b Remodels Membrane Lipids in Response to Phosphate Limitation
Source: mBio. 2022 May 16;13(3):e00247-22. doi: 10.1128/mbio.00247-22 (PMC9239053; doi:10.1128/mbio.00247-22)
Supplement: FIG S1 [file mbio.00247-22-s0003.docx]

**
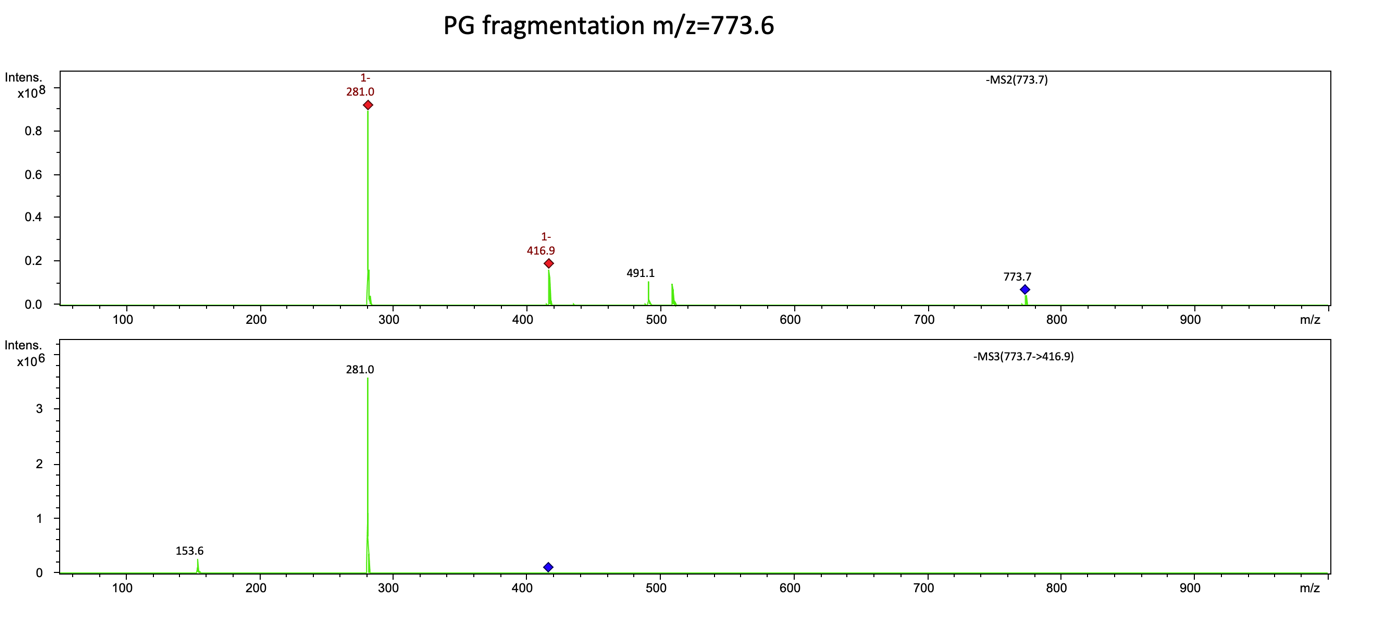
**

**Figure S1**, Fragmentation of the phosphatidylglycerol (PG) lipid *m/z* of 773.6 by MS^n^, showing the monounsaturated fatty acid C18:1 (*m/z* 281) and the formation of the characteristic ion for PG (*m/z* 153).
