# Supplementary material for: The Proteobacterial Methanotroph Methylosinus trichosporium OB3b Remodels Membrane Lipids in Response to Phosphate Limitation
Source: mBio. 2022 May 16;13(3):e00247-22. doi: 10.1128/mbio.00247-22 (PMC9239053; doi:10.1128/mbio.00247-22)
Supplement: FIG S2 [file mbio.00247-22-s0004.docx]

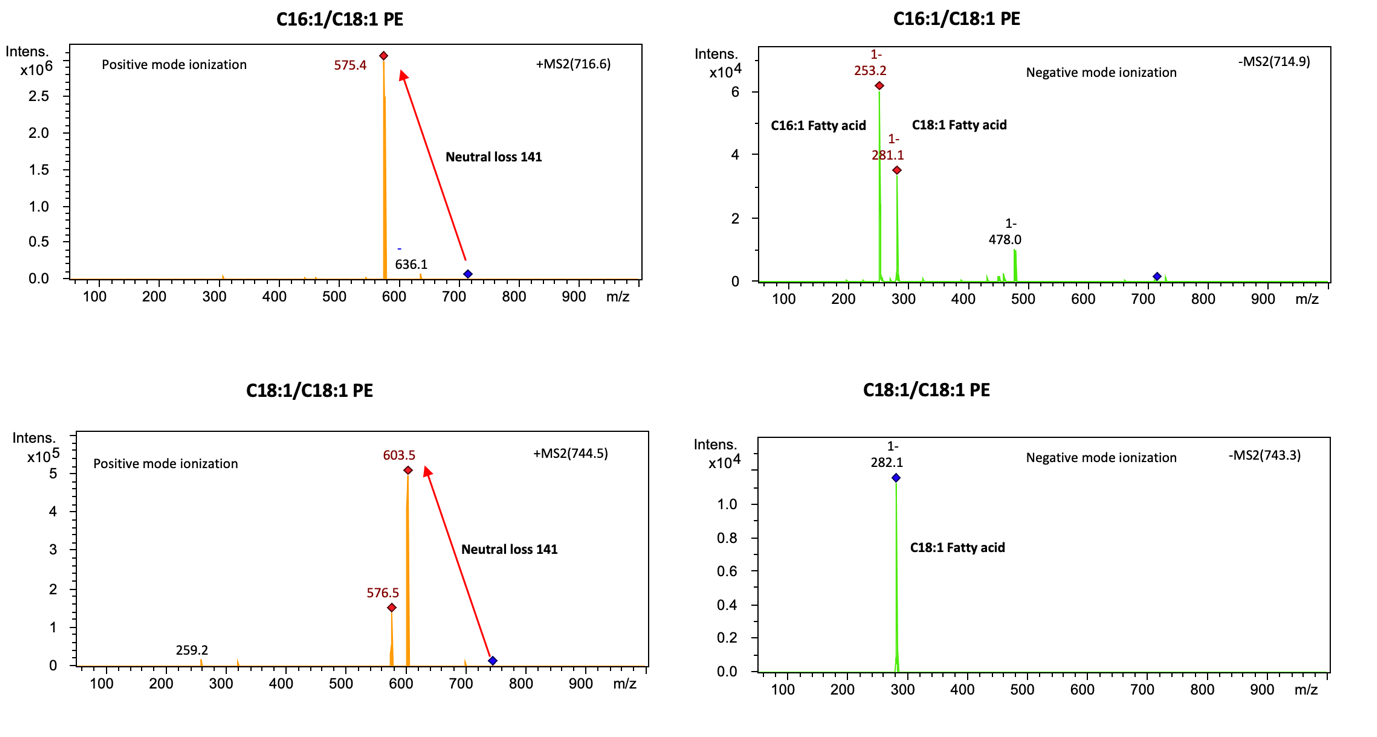
 B)


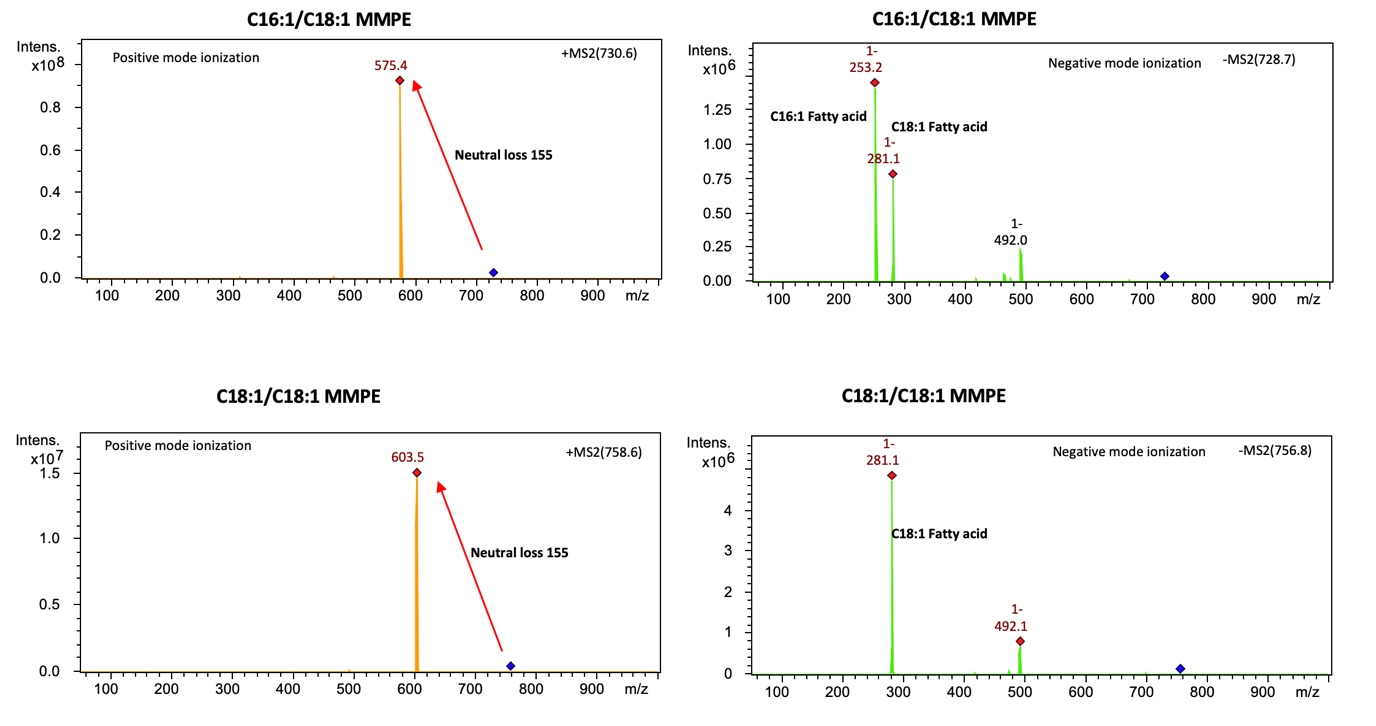
 C)


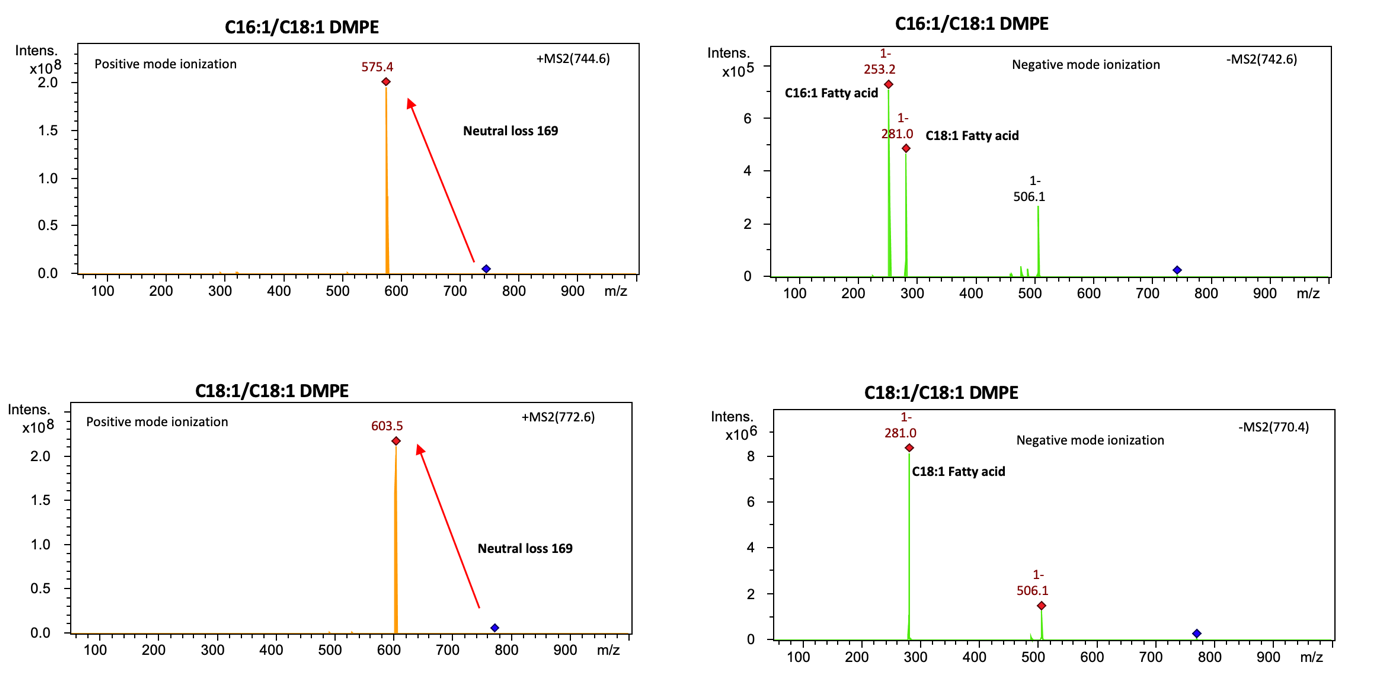


**Figure S2**, Fragmentation of the phosphatidylethanolamine (PE) (A), monomethylated PE (MMPE) (B), and demethylated PE (DMPE) (C) by MS^n^, showing characteristic neutral loss of 141, 155 and 169 respectively in the positive mode (left side). Fragmentation in the negative mode (right side) shows the corresponding monounsaturated fatty acid C18:1 and C16:1 (*m/z* 253), respectively.
