# Supplementary material for: The Proteobacterial Methanotroph Methylosinus trichosporium OB3b Remodels Membrane Lipids in Response to Phosphate Limitation
Source: mBio. 2022 May 16;13(3):e00247-22. doi: 10.1128/mbio.00247-22 (PMC9239053; doi:10.1128/mbio.00247-22)
Supplement: FIG S3 [file mbio.00247-22-s0005.docx]

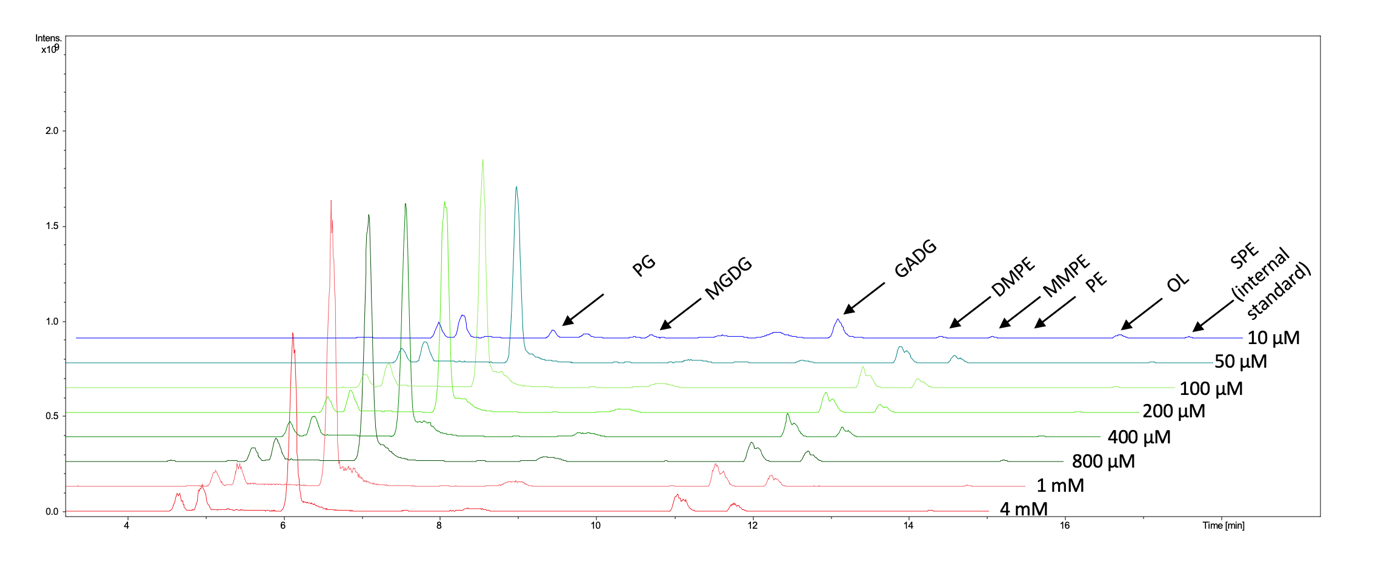


**Figure S3**, Liquid chromatography coupled with mass spectrometry (LC- MS) chromatogram showing the detection of lipids by MS from *Methylosinus trichosporium* OB3b cultivated under various phosphate levels (from 4 mM to 10 µM) in the modified NMS medium. SPE, sphingosylphosphatidylethanolamine used as internal standard. PG, phosphatidylglycerol; MGDG, monoglucosyldiacylglycerol; GADG, glucuronic acid diacylglycerol; DMPE, dimethyl-phosphatidylethanolamine; MMPE, monomethyl- phosphatidylethanolamine; PE, phosphatidylethanolamine; OL, ornithine lipids.
