# Supplementary material for: The Proteobacterial Methanotroph Methylosinus trichosporium OB3b Remodels Membrane Lipids in Response to Phosphate Limitation
Source: mBio. 2022 May 16;13(3):e00247-22. doi: 10.1128/mbio.00247-22 (PMC9239053; doi:10.1128/mbio.00247-22)
Supplement: FIG S4 [file mbio.00247-22-s0006.docx]

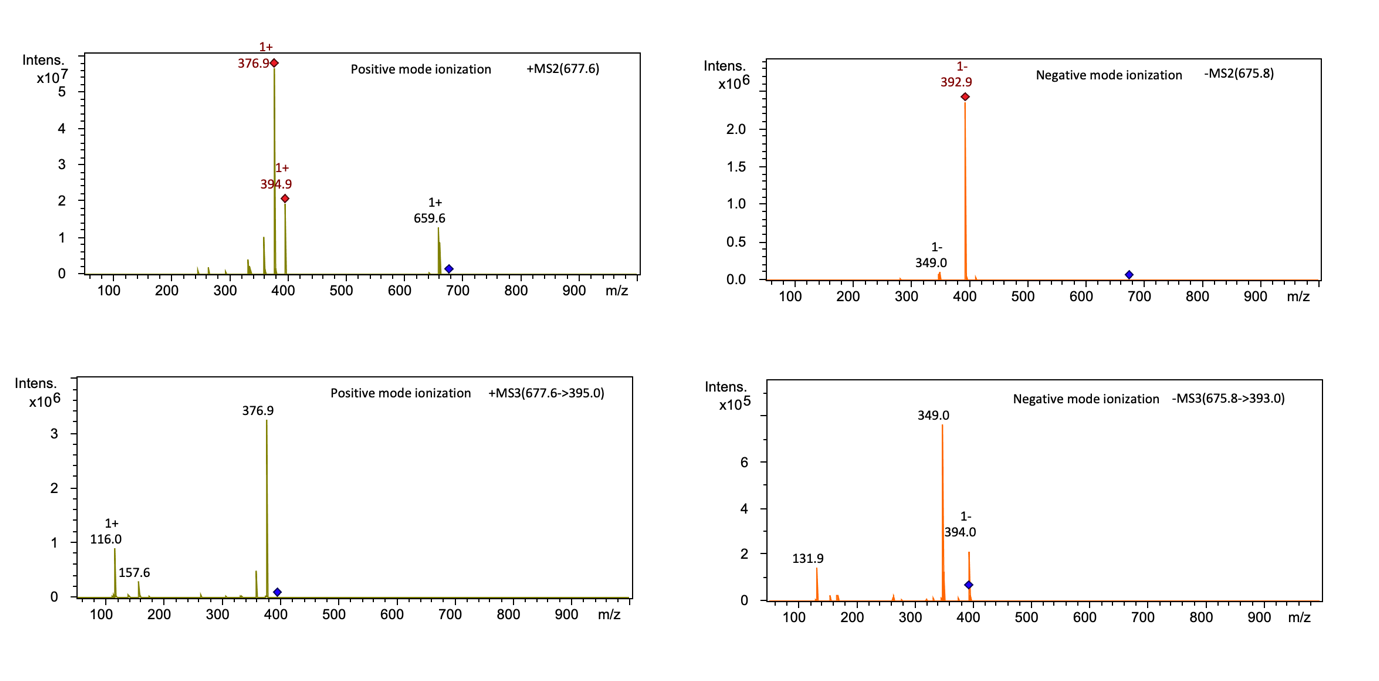


**Figure S4**, MS^n^ fragmentation of ornithine lipid produced in *Methylosinus trichosporium* OB3b in the positive mode ([M+H]^+^, *m/z* 677.6) and negative mode ([M-H]^-^, *m/z* 675.6), respectively. MS^3^ fragmentation leads to the formation of the diagnostic ion of OL in both positive mode (*m/z* 116, left side) and negative mode (*m/z* 131, right side).
