# Supplementary material for: The Proteobacterial Methanotroph Methylosinus trichosporium OB3b Remodels Membrane Lipids in Response to Phosphate Limitation
Source: mBio. 2022 May 16;13(3):e00247-22. doi: 10.1128/mbio.00247-22 (PMC9239053; doi:10.1128/mbio.00247-22)
Supplement: FIG S5 [file mbio.00247-22-s0007.docx]

1.
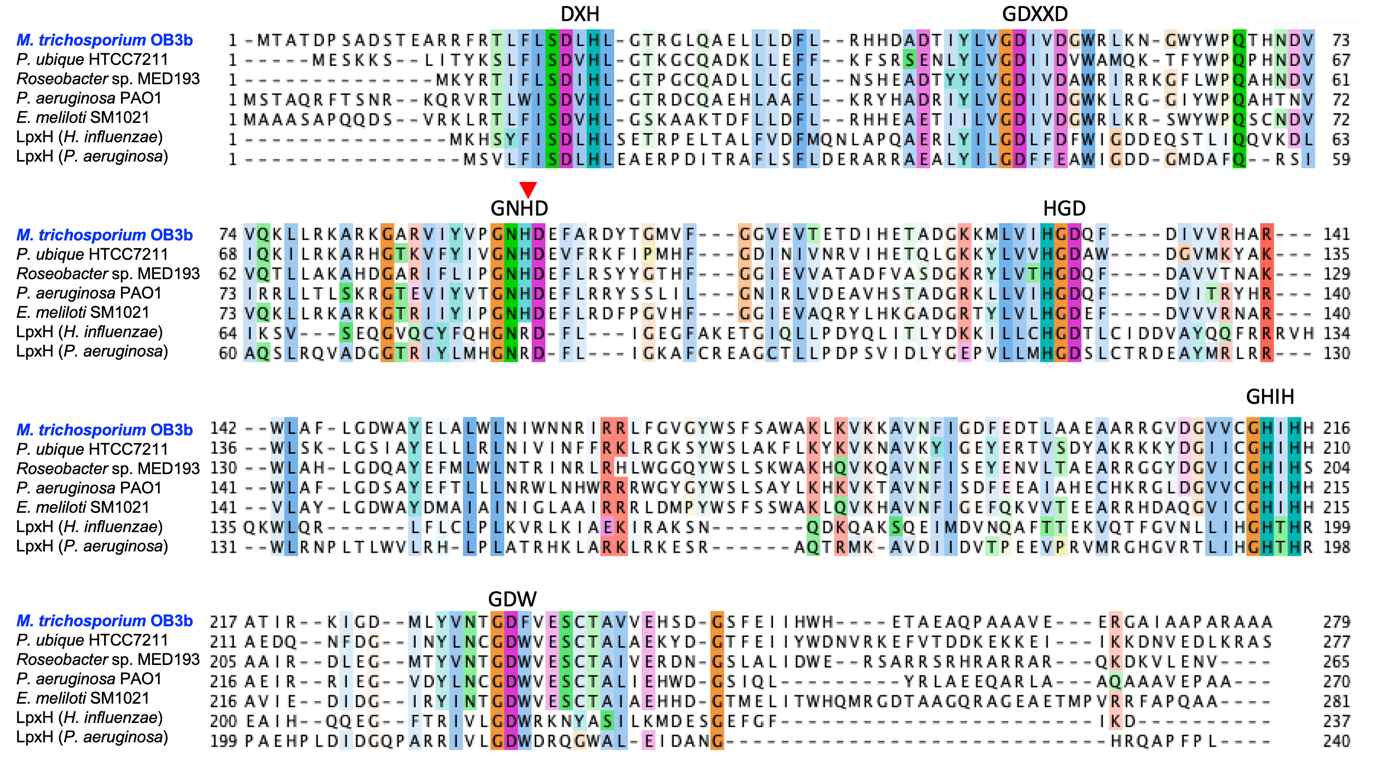

2.
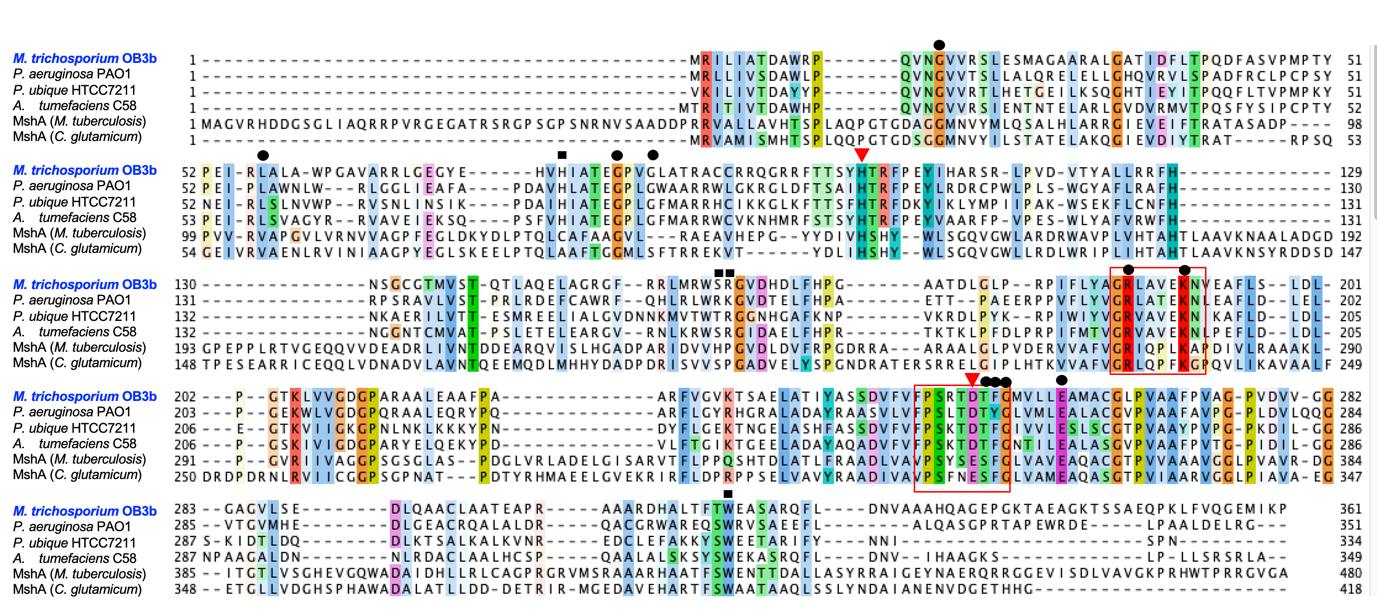


**Figure S5** Sequence alignment of PlcP (**A**) and Agt (**B**). The conserved motifs DXH, GDXXH, GNHD, HGD, GHIH and GDW found in PlcP and LpxH are shown in (**A**). The key His shown in the third motif (highlighted in a red triangle) is conserved in PlcP but not LpxH, and is required for PlcP phospholipase activity (Wei et al., 2018). The red boxes in (**B**) show conserved motifs involved in UDP-sugar binding in GT4-type glycosyltransferases and the critical His-Asp dyad for Agt activity is shown by two red arrows (Wei et al., 2021). Conserved residues interacting with UDP-sugar and diacylglycerol (DAG) are highlighted by the blacked circles and black squares, respectively.
