# Supplementary material for: The Proteobacterial Methanotroph Methylosinus trichosporium OB3b Remodels Membrane Lipids in Response to Phosphate Limitation
Source: mBio. 2022 May 16;13(3):e00247-22. doi: 10.1128/mbio.00247-22 (PMC9239053; doi:10.1128/mbio.00247-22)
Supplement: TABLE S1 [file mbio.00247-22-s0001.docx]

**Supplementary table 1 list of primers used in this study**

| **Name** | **Sequence (5’-3’)** | **Function** |
| --- | --- | --- |
| OB3bplcP’A-fwd | cacacaggaaacagctatgacatgattacgGCAAAGTTTCTCCCGCGCGC | Gibson cloning |
| OB3bplcP’A­-rev | aacagtttttatgcaCCGAGGAAGGCGAGCCAGCG | Gibson cloning |
| Gm-fwd | gctcgccttcctcggTGCATAAAAACTGTTGTAATTCATTAAGCATTC | Cloning of the Gm cassette |
| Gm-rev | gtcgacaccatggttGGCGGCGTTGTGACAATTTAC | Cloning of the Gm cassette |
| OB3bAgt'B_fwd | tgtcacaacgccgccAACCATGGTGTCGACGCAGAC | Gibson cloning |
| OB3bAgt'B_rev | actctagaggatccccgggtaccgagctcgTCACGGCTTGATCATCTCTCC | Gibson cloning |
| plcPF | CCGAGCTTCTGCTCGACTTC | Confirmation of the mutant |
| AgtR | AGACAGGCCGCCTGCAAATC | Confirmation of the mutant |
| Down Agt  CplcP_fwd  CplcP_rev  AgtF comp  pK18 KmF | AGCCGGAAGAGGAAGAGACC  CTAGAACTAGTGGATCCCCCGGGCTGCAGGCGAGCTGGCCGGTCAAGG  AGGTCGACGGTATCGATAAGCTTGATATCGGAGGAAGAGACCGCGCCC  GTGGCGTCCGCAAGTGAATG  TTGCCGCCAAGGATCTGATG | Confirmation of the mutant  Gibson cloning to complement the mutant  Gibson cloning to complement the mutant  Confirmation of the complemented mutant  Confirmation of the complemented mutant |
