# Supplementary material for: The Proteobacterial Methanotroph Methylosinus trichosporium OB3b Remodels Membrane Lipids in Response to Phosphate Limitation
Source: mBio. 2022 May 16;13(3):e00247-22. doi: 10.1128/mbio.00247-22 (PMC9239053; doi:10.1128/mbio.00247-22)
Supplement: FIG S6 [file mbio.00247-22-s0008.docx]

**
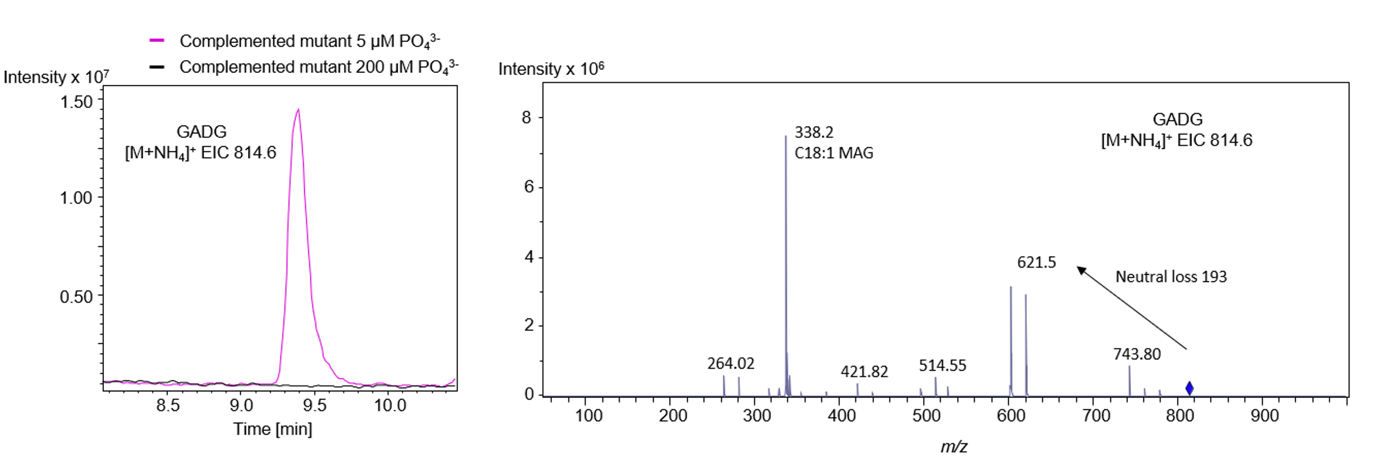
**

**
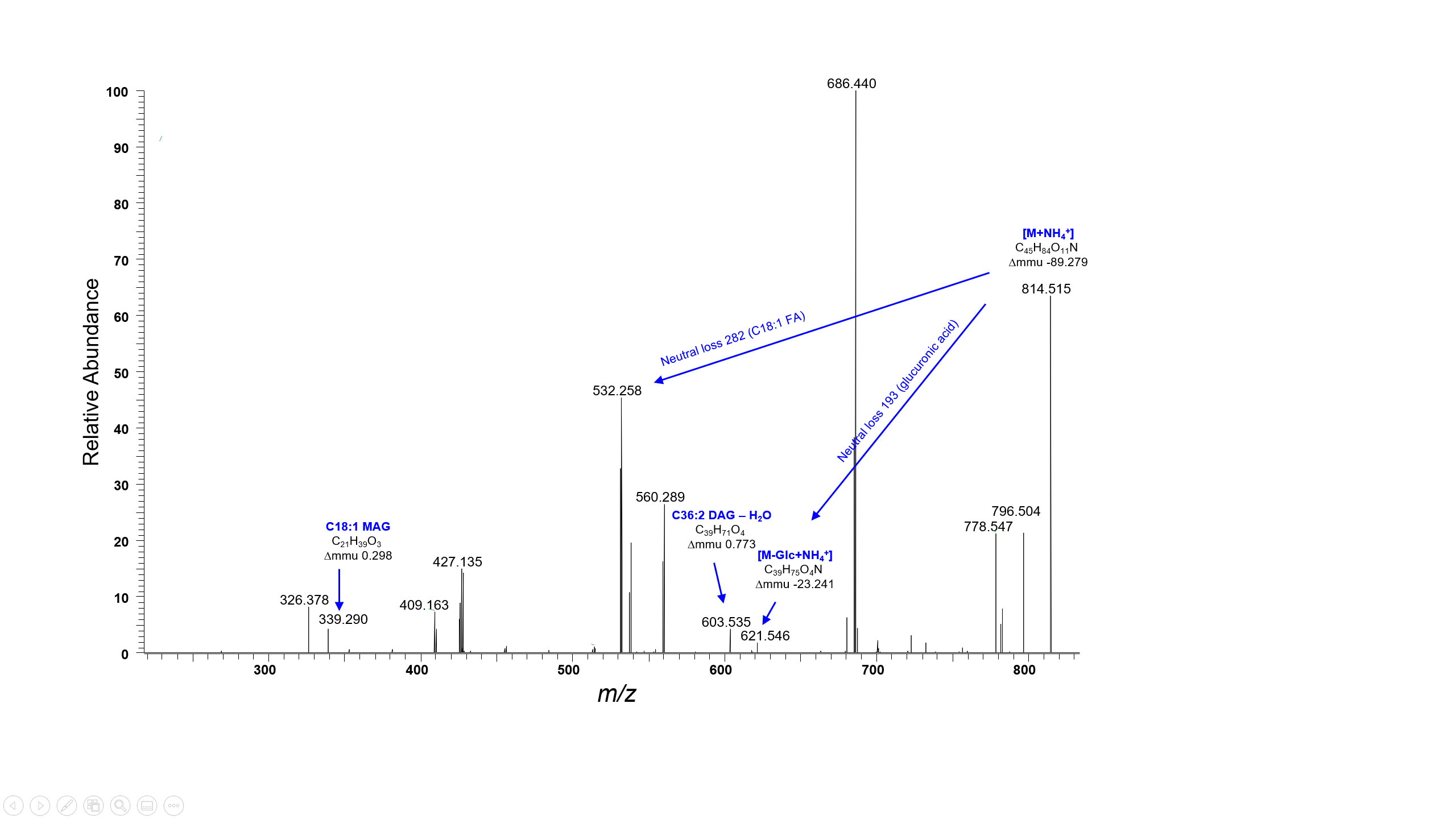
**

**Figure S6 Upper panel**, complemented mutant restored GADG lipid production in P deplete but not P replete condition. Extracted ion chromatogram (EIC) *m/z*=814.6 GADG lipid species and its fragmentation pattern in positive ionisation mode using Bruker amaZon SL. **Lower panel**, analysis of the GADG lipid [M+NH_4_^+^] *m/z*= 814.5 species by high resolution mass spectrometry using Orbitrap Fusion in the positive mode. Lipid extract from wild-type *Methylosinus trichosporium* OB3b grown in phosphate deplete condition (10 µM) was injected through direct infusion and precursor ions of *m/z* of 814.5 ± 0.5 were selected. Gas pressure 1 psi; Voltage 1.7 kV. MS^2^ fragmentation carried out by collision induced dissociation (CID) with collision energy of 32% and microscans of 10. Constant neutral loss of glucuronic acid from the parent ion was detected (*m/z* 814.515🡪 621.546) and the formation of the C36:2 diacylglycerol (DAG, *m/z* 603.535) was also observed. Further fragmentation of C36:2 DAG results in the detection of C18:1 monoacylglycerol (MAG). Formula of masses were generated using the Thermo Xcalibur and mass tolerance of 100 milli mass unit (mmu) was permitted.
