# Supplementary material for: The Proteobacterial Methanotroph Methylosinus trichosporium OB3b Remodels Membrane Lipids in Response to Phosphate Limitation
Source: mBio. 2022 May 16;13(3):e00247-22. doi: 10.1128/mbio.00247-22 (PMC9239053; doi:10.1128/mbio.00247-22)
Supplement: TABLE S2 [file mbio.00247-22-s0002.pdf]

assembly_id	NCBI_domain	NCBI_phylum	NCBI_class	NCBI_order	NCBI_family	NCBI_genus	NCBI_species
GCF_000008325.1	Bacteria	Proteobacteria	Gammaproteobacteria	Methylococcales	Methylococcaceae	Methylococcus	Methylococcus capsulatus
GCF_000019665.1	Bacteria	Verrucomicrobia	Methylacidiphilae	Methylacidiphilales	Methylacidiphilaceae	Methylacidiphilum	Methylacidiphilum infernorum
GCF_000021745.1	Bacteria	Proteobacteria	Alphaproteobacteria	Hyphomicrobiales	Beijerinckiaceae	Methylocella	Methylocella silvestris
GCF_000188155.2	Bacteria	Proteobacteria	Alphaproteobacteria	Hyphomicrobiales	Methylocystaceae	Methylocystis	Methylocystis sp. ATCC 49242
GCF_000190755.2	Bacteria	Proteobacteria	Gammaproteobacteria	Methylococcales	Methylococcaceae	Methylobacter	Methylobacter tundripaludum
GCF_000214275.2	Bacteria	Proteobacteria	Gammaproteobacteria	Methylococcales	Methylococcaceae	Methylomicrobium	Methylomicrobium album
GCF_000214665.1	Bacteria	Proteobacteria	Gammaproteobacteria	Methylococcales	Methylococcaceae	Methylomonas	Methylomonas methanica
GCF_000283235.1	Bacteria	Proteobacteria	Alphaproteobacteria	Hyphomicrobiales	Methylocystaceae	Methylocystis	Methylocystis parvus
GCF_000297415.1	Bacteria	Verrucomicrobia	Methylacidiphilae	Methylacidiphilales	Methylacidiphilaceae	Methylacidiphilum	Methylacidiphilum fumariolicum
GCF_000297615.1	Bacteria	Proteobacteria	Gammaproteobacteria	Methylococcales	Methylococcaceae	Methylococcus	Methylococcus capsulatus
GCF_000304315.1	Bacteria	Proteobacteria	Alphaproteobacteria	Hyphomicrobiales	Methylocystaceae	Methylocystis	Methylocystis sp. SC2
GCF_000341735.1	Bacteria	Proteobacteria	Gammaproteobacteria	Methylococcales	Methylococcaceae	Methylotuvimicrobium	Methylotuvimicrobium buryatense
GCF_000365425.1	Bacteria	Proteobacteria	Gammaproteobacteria	Methylococcales	Methylococcaceae	Methylomonas	Methylomonas sp. MK1
GCF_000372845.1	Bacteria	Proteobacteria	Alphaproteobacteria	Hyphomicrobiales	Methylocystaceae	Methylocystis	Methylocystis rosea
GCF_000372865.1	Bacteria	Proteobacteria	Gammaproteobacteria	Methylococcales	Methylococcaceae	Methylosarcina	Methylosarcina fibrata
GCF_000379125.1	Bacteria	Proteobacteria	Alphaproteobacteria	Hyphomicrobiales	Methylocystaceae	Methylosinus	Methylosinus sp. LW4
GCF_000383855.1	Bacteria	Proteobacteria	Gammaproteobacteria	Methylococcales	Methylococcaceae	Methylobacter	Methylobacter marinus
GCF_000384075.1	Bacteria	Proteobacteria	Gammaproteobacteria	Methylococcales	Methylococcaceae	Methylovulum	Methylovulum miyakonense
GCF_000385335.1	Bacteria	Proteobacteria	Alphaproteobacteria	Hyphomicrobiales	Beijerinckiaceae	Methyloferula	Methyloferula stellata
GCF_000421465.1	Bacteria	Proteobacteria	Gammaproteobacteria	Methylococcales	Methylothermaceae	Methylohalobius	Methylohalobius crimeensis
GCF_000424685.1	Bacteria	Proteobacteria	Gammaproteobacteria	Methylococcales	Methylococcaceae	Methylococcus	Methylococcus capsulatus
GCF_000427385.1	Bacteria	Proteobacteria	Gammaproteobacteria	Methylococcales	Methylococcaceae	Methylocaldum	Methylocaldum szegediense
GCF_000427445.1	Bacteria	Proteobacteria	Alphaproteobacteria	Hyphomicrobiales	Beijerinckiaceae	Methylocapsa	Methylocapsa acidiphila
GCF_000427625.1	Bacteria	Proteobacteria	Gammaproteobacteria	Methylococcales	Methylococcaceae	Methylobacter	Methylobacter luteus
GCF_000496735.2	Bacteria	Proteobacteria	Gammaproteobacteria	Methylococcales	Methylococcaceae	Methyloglobulus	Methyloglobulus morosus
GCF_000499825.2	Bacteria	Proteobacteria	Alphaproteobacteria	Hyphomicrobiales	Methylocystaceae	Methylocystis	Methylocystis sp. SB2
GCF_000515215.1	Bacteria	Proteobacteria	Gammaproteobacteria	Methylococcales	Methylococcaceae	Methylomonas	Methylomonas sp. 11b
GCF_000527095.1	Bacteria	Proteobacteria	Gammaproteobacteria	Methylococcales	Methylococcaceae	Methylomicrobium	Methylomicrobium lacus
GCF_000527115.1	Bacteria	Proteobacteria	Alphaproteobacteria	Hyphomicrobiales	Methylocystaceae	Methylosinus	Methylosinus sp. LW3
GCF_000685825.1	Bacteria	Proteobacteria	Alphaproteobacteria	Hyphomicrobiales	Methylocystaceae	Methylocystis	Methylocystis sp. LW5
GCF_000685925.1	Bacteria	Proteobacteria	Gammaproteobacteria	Methylococcales	Methylococcaceae	Methylobacter	Methylobacter tundripaludum
GCF_000733835.1	Bacteria	Proteobacteria	Gammaproteobacteria	Methylococcales	Methylococcaceae	Methylobacter	Methylobacter tundripaludum
GCF_000733855.1	Bacteria	Proteobacteria	Gammaproteobacteria	Methylococcales	Methylococcaceae	Methylomicrobium	Methylomicrobium agile
GCF_000733935.1	Bacteria	Proteobacteria	Gammaproteobacteria	Methylococcales	Methylococcaceae	Methylomarinum	Methylomarinum vadi
GCF_000745215.1	Bacteria	Proteobacteria	Alphaproteobacteria	Hyphomicrobiales	Methylocystaceae	Methylosinus	Methylosinus sp. PW1
GCF_000746085.1	Bacteria	Proteobacteria	Alphaproteobacteria	Hyphomicrobiales	Beijerinckiaceae	Methylocapsa	Methylocapsa aurea
GCF_000746145.1	Bacteria	Proteobacteria	Gammaproteobacteria	Methylococcales	Methylococcaceae	Methylobacter	Methylobacter sp. BBA5.1
GCF_000785705.2	Bacteria	Proteobacteria	Gammaproteobacteria	Methylococcales	Methylococcaceae	Methylomonas	Methylomonas denitrificans
GCF_000817245.1	Bacteria	Verrucomicrobia	Methylacidiphilae	Methylacidiphilales	Methylacidiphilaceae	Methylacidiphilum	Methylacidiphilum kamchatkense
GCF_000934725.1	Bacteria	Proteobacteria	Gammaproteobacteria	Methylococcales	Methylococcaceae	Methyloterricola	Methyloterricola oryzae
GCF_000963695.1	Bacteria	Proteobacteria	Gammaproteobacteria	Methylococcales	Methylococcaceae	Methylocucumis	Methylocucumis oryzae
GCF_000968535.2	Bacteria	Proteobacteria	Gammaproteobacteria	Methylococcales	Methylococcaceae	Methylotuvimicrobium	Methylotuvimicrobium alcaliphilum
GCF_001312345.1	Bacteria	Proteobacteria	Gammaproteobacteria	Methylococcales	Methylococcaceae	Methylogaea	Methylogaea oryzae
GCF_001644015.1	Bacteria	Proteobacteria	Gammaproteobacteria	Methylococcales	Methylococcaceae	Methylomonas	Methylomonas lenta
GCF_001644025.1	Bacteria	Proteobacteria	Gammaproteobacteria	Methylococcales	Methylococcaceae	Methylomonas	Methylomonas koyamae
GCF_001644035.1	Bacteria	Proteobacteria	Gammaproteobacteria	Methylococcales	Methylococcaceae	Methylomonas	Methylomonas methanica
GCF_001644095.1	Bacteria	Proteobacteria	Gammaproteobacteria	Methylococcales	Methylococcaceae	Methylomonas	Methylomonas koyamae
GCF_001644115.1	Bacteria	Proteobacteria	Gammaproteobacteria	Methylococcales	Methylococcaceae	Methylomonas	Methylomonas methanica
GCF_001644125.1	Bacteria	Proteobacteria	Alphaproteobacteria	Hyphomicrobiales	Methylocystaceae	Methylosinus	Methylosinus sp. R-45379
GCF_001644135.1	Bacteria	Proteobacteria	Gammaproteobacteria	Methylococcales	Methylococcaceae	Methylomonas	Methylomonas koyamae
GCF_001644685.1	Bacteria	Proteobacteria	Gammaproteobacteria	Methylococcales	Methylococcaceae	Methylomonas	Methylomonas sp. DH-1
GCF_001675455.1	Bacteria	Proteobacteria	Alphaproteobacteria	Hyphomicrobiales	Methylocystaceae	Methylosinus	Methylosinus sp. 3S-1
GCF_001856095.1	Bacteria	Proteobacteria	Gammaproteobacteria	Methylococcales	Methylococcaceae	Methylomonas	Methylomonas sp. LWB
GCF_002005105.1	Bacteria	Proteobacteria	Gammaproteobacteria	Methylococcales	Methylococcaceae	Methylocaldum	Methylocaldum sp. 14B
GCF_002072955.1	Bacteria	Proteobacteria	Gammaproteobacteria	Methylococcales	Methylococcaceae	Methyloprofundus	Methyloprofundus sedimenti
GCF_002117405.1	Bacteria	Proteobacteria	Alphaproteobacteria	Hyphomicrobiales	Methylocystaceae	Methylocystis	Methylocystis bryophila
GCF_002127725.1	Bacteria	Proteobacteria	Gammaproteobacteria	Methylococcales	Methylococcaceae	Methylocaldum	Methylocaldum sp. SAD2
GCF_002209385.1	Bacteria	Proteobacteria	Gammaproteobacteria	Methylococcales	Methylococcaceae	Methylovulum	Methylovulum psychrotolerans
GCF_002441955.1	Bacteria	Proteobacteria	Gammaproteobacteria	Methylococcales	Methylococcaceae	Methylomonas	Methylomonas koyamae
GCF_002752655.1	Bacteria	Proteobacteria	Alphaproteobacteria	Hyphomicrobiales	Methylocystaceae	Methylosinus	Methylosinus trichosporium
GCF_002834115.1	Bacteria	Proteobacteria	Gammaproteobacteria	Methylococcales	Methylococcaceae	Methylomonas	Methylomonas sp. Kb3
GCF_002891535.1	Bacteria	Proteobacteria	Alphaproteobacteria	Hyphomicrobiales	Beijerinckiaceae	Methylocella	Methylocella silvestris
GCF_002923755.1	Bacteria	Proteobacteria	Gammaproteobacteria	Methylococcales	Methylococcaceae	Methylovulum	Methylovulum psychrotolerans
GCF_002934365.1	Bacteria	Proteobacteria	Gammaproteobacteria	Methylococcales	Methylococcaceae	Methylobacter	Methylobacter tundripaludum
GCF_002934385.1	Bacteria	Proteobacteria	Gammaproteobacteria	Methylococcales	Methylococcaceae	Methylobacter	Methylobacter tundripaludum
GCF_003113245.1	Bacteria	Proteobacteria	Alphaproteobacteria	Hyphomicrobiales	Methylocystaceae	Methylocystis	Methylocystis sp. MitZ-2018
GCF_003113265.1	Bacteria	Proteobacteria	Alphaproteobacteria	Hyphomicrobiales	Methylocystaceae	Methylosinus	Methylosinus sporium
GCF_003584645.1	Bacteria	Proteobacteria	Gammaproteobacteria	Methylococcales	Methylococcaceae	Methylocaldum	Methylocaldum marinum
GCF_003722355.1	Bacteria	Proteobacteria	Alphaproteobacteria	Hyphomicrobiales	Methylocystaceae	Methylocystis	Methylocystis hirsuta
GCF_003855495.1	Bacteria	Proteobacteria	Alphaproteobacteria	Hyphomicrobiales	Methylocystaceae	Methylocystis	Methylocystis rosea
GCF_003932755.1	Bacteria	Proteobacteria	Gammaproteobacteria	Methylococcales	Methylococcaceae	Methylomicrobium	Methylomicrobium sp. wino1
GCF_003994235.2	Bacteria	Proteobacteria	Gammaproteobacteria	Methylococcales	Methylococcaceae	Methylobacter	Candidatus Methylobacter oryzae
GCF_004343025.1	Bacteria	Proteobacteria	Gammaproteobacteria	Methylococcales	Methylococcaceae	Methylomonas	Methylomonas methanica
GCF_004366495.1	Bacteria	Proteobacteria	Alphaproteobacteria	Hyphomicrobiales	Methylocystaceae	Methylosinus	Methylosinus sp. sav-2
GCF_004421155.1	Bacteria	Verrucomicrobia	Methylacidiphilae	Methylacidiphilales	Methylacidiphilaceae	Methylacidiphilum	Methylacidiphilum fumariolicum
GCF_004421165.1	Bacteria	Verrucomicrobia	Methylacidiphilae	Methylacidiphilales	Methylacidiphilaceae	Methylacidiphilum	Methylacidiphilum fumariolicum
GCF_004421175.1	Bacteria	Verrucomicrobia	Methylacidiphilae	Methylacidiphilales	Methylacidiphilaceae	Methylacidiphilum	Methylacidiphilum fumariolicum
GCF_004421185.1	Bacteria	Verrucomicrobia	Methylacidiphilae	Methylacidiphilales	Methylacidiphilaceae	Methylacidiphilum	Methylacidiphilum sp. Yel
GCF_004421195.1	Bacteria	Verrucomicrobia	Methylacidiphilae	Methylacidiphilales	Methylacidiphilaceae	Methylacidiphilum	Methylacidiphilum fumariolicum
GCF_004421255.1	Bacteria	Verrucomicrobia	Methylacidiphilae	Methylacidiphilales	Methylacidiphilaceae	Methylacidiphilum	Methylacidiphilum sp. Phi
GCF_004802635.2	Bacteria	Proteobacteria	Alphaproteobacteria	Hyphomicrobiales	Methylocystaceae	Methylocystis	Methylocystis heyeri
GCF_005771425.1	Bacteria	Proteobacteria	Alphaproteobacteria	Hyphomicrobiales	Methylocystaceae	Methylocystis	Methylocystis sp. B8
GCF_005931095.1	Bacteria	Proteobacteria	Gammaproteobacteria	Methylococcales	Methylococcaceae	Methylotuvimicrobium	Methylotuvimicrobium buryatense
GCF_006175985.1	Bacteria	Proteobacteria	Gammaproteobacteria	Methylococcales	Methylococcaceae	Methylotetracoccus	Methylotetracoccus oryzae

GCF_006483455.1	Bacteria	Proteobacteria	Gammaproteobacteria	Methylococcales	Methylococcaceae	Methylomonas	Methylomonas koyamae
GCF_007004125.1	Bacteria	Proteobacteria	Alphaproteobacteria	Hyphomicrobiales	Methylocystaceae	Methylosinus	Methylosinus sporium
GCF_008632455.1	Bacteria	Proteobacteria	Gammaproteobacteria	Methylococcales	Methylococcaceae	Methylomonas	Methylomonas rhizoryzae
GCF_009498235.1	Bacteria	Proteobacteria	Gammaproteobacteria	Methylococcales	Methylococcaceae	Candidatus Methylospira	Candidatus Methylospira mobilis
GCF_009685175.1	Bacteria	Proteobacteria	Alphaproteobacteria	Hyphomicrobiales	Methylocystaceae	Methylocystis	Methylocystis rosea
GCF_009685195.1	Bacteria	Proteobacteria	Alphaproteobacteria	Hyphomicrobiales	Methylocystaceae	Methylocystis	Methylocystis parvus
GCF_009811655.1	Bacteria	Proteobacteria	Alphaproteobacteria	Hyphomicrobiales	Methylocystaceae	Methylosinus	Methylosinus sp. Ce-a6
GCF_009828925.1	Bacteria	Proteobacteria	Gammaproteobacteria	Methylococcales	Methylococcaceae	Methylicorpusculum	Methylicorpusculum oleiharenae
GCF_009936375.1	Bacteria	Proteobacteria	Alphaproteobacteria	Hyphomicrobiales	Methylocystaceae	Methylosinus	Methylosinus sp. C49
GCF_011058845.1	Bacteria	Proteobacteria	Alphaproteobacteria	Hyphomicrobiales	Methylocystaceae	Methylocystis	Methylocystis sp. MJC1
GCF_011378635.1	Bacteria	Proteobacteria	Alphaproteobacteria	Hyphomicrobiales	Methylocystaceae	Methylosinus	Methylosinus sp. RM1
GCF_012769535.1	Bacteria	Proteobacteria	Gammaproteobacteria	Methylococcales	Methylococcaceae	Methylococcus	Methylococcus sp. IM1
GCF_013141865.1	Bacteria	Proteobacteria	Gammaproteobacteria	Methylococcales	Methylococcaceae	Methylomonas	Methylomonas sp. ZR1
GCF_013350005.1	Bacteria	Proteobacteria	Alphaproteobacteria	Hyphomicrobiales	Methylocystaceae	Methylocystis	Methylocystis sp. FS
GCF_014850955.1	Bacteria	Proteobacteria	Gammaproteobacteria	Methylococcales	Methylococcaceae	Methylomonas	Methylomonas sp. EbA
GCF_014850975.1	Bacteria	Proteobacteria	Gammaproteobacteria	Methylococcales	Methylococcaceae	Methylomonas	Methylomonas sp. EbB
GCF_015476545.1	Bacteria	Proteobacteria	Gammaproteobacteria	Methylococcales	Methylococcaceae	Methylobacter	Methylobacter sp. BlB1
GCF_015709455.1	Bacteria	Proteobacteria	Alphaproteobacteria	Hyphomicrobiales	Methylocystaceae	Methylosinus	Methylosinus sp. H3A
GCF_015709465.1	Bacteria	Proteobacteria	Alphaproteobacteria	Hyphomicrobiales	Methylocystaceae	Methylocystis	Methylocystis sp. H15
GCF_015709515.1	Bacteria	Proteobacteria	Alphaproteobacteria	Hyphomicrobiales	Methylocystaceae	Methylocystis	Methylocystis sp. H62
GCF_015709525.1	Bacteria	Proteobacteria	Alphaproteobacteria	Hyphomicrobiales	Methylocystaceae	Methylocystis	Methylocystis sp. L43
GCF_015709545.1	Bacteria	Proteobacteria	Alphaproteobacteria	Hyphomicrobiales	Methylocystaceae	Methylocystis	Methylocystis sp. H4A
GCF_015711015.1	Bacteria	Proteobacteria	Gammaproteobacteria	Methylococcales	Methylococcaceae	Methylomonas	Methylomonas sp. LL1
GCF_016106025.1	Bacteria	Proteobacteria	Gammaproteobacteria	Methylococcales	Methylococcaceae	Methylococcus	Methylococcus sp. BF19-07
GCF_016745395.1	Bacteria	Proteobacteria	Alphaproteobacteria	Hyphomicrobiales	Methylocystaceae	Methylocystis	Methylocystis sp. Sn-Cys
GCF_016745425.1	Bacteria	Proteobacteria	Gammaproteobacteria	Methylococcales	Methylococcaceae	Methylomicrobium	Methylomicrobium sp. RS1
GCF_016865255.1	Bacteria	Proteobacteria	Gammaproteobacteria	Methylococcales	Methylococcaceae	Methylomonas	Methylomonas sp. LW13
GCF_016925495.1	Bacteria	Proteobacteria	Gammaproteobacteria	Methylococcales	Methylococcaceae	Methylococcus	Methylococcus sp. EFPC2
GCF_016925515.1	Bacteria	Proteobacteria	Gammaproteobacteria	Methylococcales	Methylococcaceae	Methylomonas	Methylomonas sp. EFPC1
GCF_017310505.1	Bacteria	Verrucomicrobia	Methylacidiphilae	Methylacidiphilales	Methylacidiphilaceae	Methylacidiphilum	Methylacidiphilum sp. IT6
GCF_017310525.1	Bacteria	Verrucomicrobia	Methylacidiphilae	Methylacidiphilales	Methylacidiphilaceae	Methylacidiphilum	Methylacidiphilum sp. IT5
GCF_017310545.1	Bacteria	Verrucomicrobia	NA	NA	NA	Methylacidimicrobium	Methylacidimicrobium sp. B4
GCF_017832375.1	Bacteria	Proteobacteria	Gammaproteobacteria	Methylococcales	Methylococcaceae	Methylocaldum	Methylocaldum sp. RMAD-M
GCF_018722605.1	Bacteria	Proteobacteria	Gammaproteobacteria	Methylococcales	Methylococcaceae	Methylovulum	Methylovulum psychrotolerans
GCF_018734325.1	Bacteria	Proteobacteria	Gammaproteobacteria	Methylococcales	Methylococcaceae	Methylomonas	Methylomonas paludis
GCF_018887255.1	Bacteria	Proteobacteria	Alphaproteobacteria	Hyphomicrobiales	Methylocystaceae	Methylosinus	Methylosinus sp. KRF6
GCF_019285515.1	Bacteria	Proteobacteria	Gammaproteobacteria	Methylococcales	Methylococcaceae	Methylococcus	Methylococcus sp. Mc7
GCF_019285535.1	Bacteria	Proteobacteria	Gammaproteobacteria	Methylococcales	Methylococcaceae	Methylococcus	Methylococcus capsulatus
GCF_019285555.1	Bacteria	Proteobacteria	Gammaproteobacteria	Methylococcales	Methylococcaceae	Methylococcus	Methylococcus capsulatus
GCF_019285575.1	Bacteria	Proteobacteria	Gammaproteobacteria	Methylococcales	Methylococcaceae	Methylococcus	Methylococcus capsulatus
GCF_019429645.1	Bacteria	Verrucomicrobia	Methylacidiphilae	Methylacidiphilales	Methylacidiphilaceae	Methylacidiphilum	Methylacidiphilum fumariolicum
GCF_900114285.1	Bacteria	Proteobacteria	Alphaproteobacteria	Hyphomicrobiales	Beijerinckiaceae	Methylocapsa	Methylocapsa palsarum
GCF_900155475.1	Bacteria	Proteobacteria	Gammaproteobacteria	Methylococcales	Methylococcaceae	Methylomagnum	Methylomagnum ishizawai
GCF_900163745.1	Bacteria	Proteobacteria	Gammaproteobacteria	Methylococcales	Crenotrichaceae	Crenothrix	Crenothrix polyspora
GCF_901905185.1	Bacteria	Proteobacteria	Alphaproteobacteria	Hyphomicrobiales	Beijerinckiaceae	Methylocella	Methylocella tundrae
GCF_902143375.2	Bacteria	Verrucomicrobia	NA	NA	NA	Methylacidimicrobium	Methylacidimicrobium tartarophylax
GCF_902143385.2	Bacteria	Verrucomicrobia	NA	NA	NA	Methylacidimicrobium	Methylacidimicrobium cyclopophantes
GCF_902806695.1	Bacteria	Proteobacteria	Gammaproteobacteria	Methylococcales	Methylococcaceae	Methylobacter	Candidatus Methylobacter favarea
GCF_903064525.1	Bacteria	Verrucomicrobia	NA	NA	NA	Methylacidimicrobium	Methylacidimicrobium sp. AP8
GCF_903064685.1	Bacteria	Proteobacteria	Gammaproteobacteria	Methylococcales	Methylococcaceae	Methylomonas	Methylomonas sp. EbB
GCF_903064715.1	Bacteria	Proteobacteria	Gammaproteobacteria	Methylococcales	Methylococcaceae	Methylomonas	Methylomonas sp. EbA
GCF_006788925.1	Bacteria	Proteobacteria	Gammaproteobacteria	Methylococcales	Methylococcaceae	Methylomicrobium	Methylomicrobium kenyense
GCF_000745375.1	Bacteria	Proteobacteria	Gammaproteobacteria	Methylococcales	Methylococcaceae	Methylobacter	Methylobacter whittenburyi
GCF_004363855.1	Bacteria	Proteobacteria	Gammaproteobacteria	Methylococcales	Methylococcaceae	Methylocaldum	Methylocaldum sp. 0917
GCF_017833335.1	Bacteria	Proteobacteria	Gammaproteobacteria	Methylococcales	Methylococcaceae	Methylocaldum	Methylocaldum sp. S3v3
GCF_017833355.1	Bacteria	Proteobacteria	Gammaproteobacteria	Methylococcales	Methylococcaceae	Methylocaldum	Methylocaldum sp. YM2
**GCA_002890675.1**	Bacteria	Proteobacteria	Alphaproteobacteria	Hyphomicrobiales	Beijerinckiaceae	NA	**Beijerinckiaceae bacterium USCa_MF**
**GCF_004564215.1**	Bacteria	Proteobacteria	Alphaproteobacteria	Rhizobiales	Beijerinckiaceae	NA	**Beijerinckiaceae bacterium MG08**
**GCA_002007425.1**	Bacteria	Proteobacteria	Gammaproteobacteria	Chromatiales	NA	NA	**Chromatiales bacterium USCg_Taylor**
